# Physical and virtual carbon metabolism of global cities

**DOI:** 10.1038/s41467-019-13757-3

**Published:** 2020-01-10

**Authors:** Shaoqing Chen, Bin Chen, Kuishuang Feng, Zhu Liu, Neil Fromer, Xianchun Tan, Ahmed Alsaedi, Tasawar Hayat, Helga Weisz, Hans Joachim Schellnhuber, Klaus Hubacek

**Affiliations:** 1 State Key Joint Laboratory of Environment Simulation and Pollution Control, School of Environment, Beijing Normal University, Beijing, 100875 China; 2 School of Environmental Science and Engineering, Sun Yat-sen University, Guangzhou, 510275 China; 3 Guangdong Provincial Key Laboratory of Environmental Pollution Control and Remediation Technology (Sun Yat-sen University), Guangzhou, 510275 China; 4 Department of Geographical Sciences, University of Maryland, College Park, MD 20742 USA; 5 Ministry of Education Key Laboratory for Earth System Modeling, Department of Earth System Science, Tsinghua University, Beijing, 100084 China; 6 Resnick Sustainability Institute, California Institute of Technology, Pasadena, CA 91125 USA; 7 Institutes of Science and Development, Chinese Academy of Sciences, Beijing, China; 8 NAAM Research Group, Faculty of Science, King Abdulaziz University, Jeddah, 21589 Saudi Arabia; 9 Department of Mathematics, Quaid-I-Azam University, Islamabad, 44000 Pakistan; 10 Potsdam Institute for Climate Impact Research, Potsdam, 14473 Germany; 11 Department of Cultural History & Theory and Department of Social Sciences, Humboldt-University Berlin, Unter den Linden 6, D-10117 Berlin, Germany; 12 Center for Energy and Environmental Sciences (IVEM), Energy and Sustainability Research Institute Groningen (ESRIG), University of Groningen, Groningen, 9747 AG The Netherlands; 13 International Institute for Applied Systems Analysis, Schlossplatz 1-A-2361, Laxenburg, Austria; 14 Department of Environmental Studies, Masaryk University, Brno, Czech Republic

**Keywords:** Climate-change policy, Energy and society

## Abstract

Urban activities have profound and lasting effects on the global carbon balance. Here we develop a consistent metabolic approach that combines two complementary carbon accounts, the physical carbon balance and the fossil fuel-derived gaseous carbon footprint, to track carbon coming into, being added to urban stocks, and eventually leaving the city. We find that over 88% of the physical carbon in 16 global cities is imported from outside their urban boundaries, and this outsourcing of carbon is notably amplified by virtual emissions from upstream activities that contribute 33–68% to their total carbon inflows. While 13–33% of the carbon appropriated by cities is immediately combusted and released as CO_2_, between 8 and 24% is stored in durable household goods or becomes part of other urban stocks. Inventorying carbon consumed and stored for urban metabolism should be given more credit for the role it can play in stabilizing future global climate.

## Abstract (web summary)

Activities in cities are important drivers of global carbon fluxes. Here the authors trace the carbon metabolism in 16 global cities in terms of both physical and virtual carbon inflows, stock changes and outflows in relation to the supply chains of urban production and consumption and show that the total carbon impacts of global cities are found to be highly varied in either per capita, intensity or density measures.

## Introduction

At present, more than half of the global population resides in cities^1^. Cities are important real-life observatories that provide opportunities to study how human activities influence global biogeochemical cycles^2,3^. Carbon, as an essential input to the economy, is found in fossil fuels, biomass, food, construction materials for buildings and infrastructure, and all sorts of products that are concentrated in cities. Carbon flows associated with cities need to be systematically measured and modeled to decrease the huge impact of urban activities on global climate^4–6^.

To date, the inventories of carbon flows of cities have concentrated on gaseous emissions. A territorial inventory is often used by local authorities to report CO_2_ emissions from within cities (e.g., refs. ^7,8^). When accounting for urban carbon footprints, flows of carbon both within and across urban territories are often considered because of cities’ high demands for goods and services from other regions^9,10^. The importance of appropriate definitions of system boundaries in carbon accounting has been emphasized in the Greenhouse Gas Protocol proposed by Local Governments for Sustainability (ICLEI), the World Resources Institute (WRI), and the C40 Cities Climate Leadership Group^11,12^. Considering both territorial and upstream (virtual) carbon, they identified three scopes of community-scale greenhouse gas (GHG) emissions: emissions within the administrative boundary of a community (Scope 1); energy-related embodied emissions outside the community boundary, such as purchase of electricity from outside of a city (Scope 2); and all other upstream emissions outside the city as a result of activities within a community’s boundary (Scope 3). Several complementary (and partially overlapping) frameworks have been proposed to measure the carbon footprints of cities, such as community-wide infrastructure footprint^13–15^, consumption-based footprint^16–18^, and wider production-based footprint^19,20^. Hybrid approaches of material flow analysis (MFA) and life-cycle analysis (LCA) have been used to account for carbon emissions associated with key urban materials or activities (e.g., ^14,21–23^). In addition, input–output analysis (IOA) has been increasingly used in urban carbon inventories due to its capability of effectively capturing emissions embodied in supply chains^16,19,24^.

On the other hand, physical carbon stocks^25,26^, natural sinks^27,28^, and fluxes in products^29^ within urban settlements have been examined in order to understand the role played by cities in the global carbon cycle, relevant to the carbon models focusing on sources and sinks in biogeochemical cycles developed at country level^30,31^. The carbon balance of urban economy is usually investigated through imports, exports, and storage of products and assets. Analyses of urban carbon flows are closely linked to the dynamics of climate change since over time, most carbon products stored in cities will eventually become waste^25^ and later on partially released as gaseous emissions^32,33^. Several studies have highlighted the importance of managing material stocks in urban areas^34,35^, but there is still a lack of detailed quantification of these urban stocks relative to the entire carbon balance and how they can contribute to future reduction of carbon emissions. A national-scale inventory by Peters et al.^36^ quantified the CO_2_ related to international trade, including emissions embodied in import and physical carbon present in various products. Similar information at city scale is missing and the contribution urban stocks can make for decarbonization is still unclear.

In this paper, we develop an integrated approach to model the urban carbon flows of 16 global cities. In most previous studies, physical carbon account and urban carbon footprint were kept separate, albeit the existence of hybrid inventories for carbon cycle^30^ and material metabolism^37^ at national scale. Here, we consider urban carbon metabolism as a whole, where physical carbon account and fossil fuel-derived gaseous carbon footprint are combined, through examining the metabolic flows of both physical carbon direct input to a city and fossil-fuel derived virtual carbon associated with upstream supply chains over a one-year accounting period. Physical carbon refers to the real carbon content in materials and products that is directly consumed, transformed or re-exported by an urban economy (see similar definitions in refs. ^25,36^). These flows include gaseous emissions (in this case, CO_2_) as well as physical carbon trapped in products that can be emitted during their lifetimes. This physical carbon is contained in biogenic products such as food and fiber as well as fossil fuels. Virtual carbon refers to fossil-fuel derived CO_2_ that was emitted in upstream supply chains of electricity and other goods and services imported to a city (see similar definitions in refs. ^38,39^), and it excludes CH_4_ and other GHGs embodied in agriculture, which can be significant. As such, fossil-fuel related physical and virtual carbon together do not cover all GHGs related to a city, and the remaining carbon in materials (e.g., wood burning fuels) may or may not considered as a net GHG in other studies (e.g., ^31,40^).

By applying this approach to 16 global cities, we find there is a wide variation in the total carbon appropriated by urban economies, in which both physical carbon and fossil-fuel derived virtual carbon play an important role. It is difficult or undesirable to generate a one-size-fits-all carbon mitigation approach for all cities due to significant differences in income level, urban form and infrastructure scale. But cities do share a need of managing their stocks, as our model shows the carbon stored as durable household goods or stocks in industrial sectors amounts to 8–24% of their total carbon inflows, comparable to carbon that already ends up as gaseous emissions for energy uses. These carbon stocks, especially those linked to investment in housing, production facilities and infrastructure will shape future emissions and could compromise on-going climate change mitigation efforts. Portraying how carbon is appropriated and stored in cities may offer an often overlooked policy option for a deep urban decarbonization, and this is unlikely to be gained from separate accounting of physical carbon balances and fossil fuel-derived carbon footprints.

## Results

### Per capita carbon inflow of cities

**Fig. 1:**
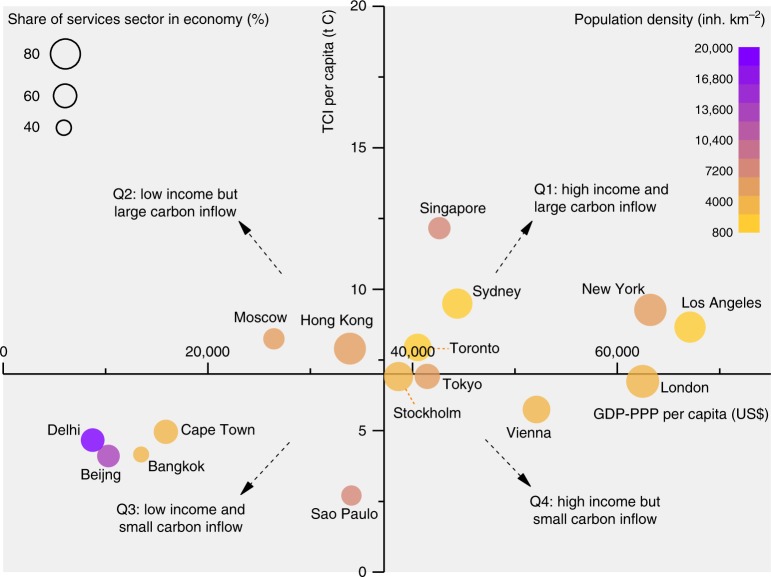
Distribution of 16 global cities by per capita total carbon inflow (TCI) and per capita GDP-PPP. TCI encapsulates both the physical carbon inflow of a city as well as the fossil fuel combustion-related virtual carbon emissions associated with a city’s upstream supply chains. Note that the modeling of virtual carbon of Beijing, Hong Kong, Singapore, London, Sydney, New York, and Los Angeles is based on city-level input–output tables, while those of the remaining cities use downscaled tables of the national economy adjusted by location quotients, as described in Methods. We use per capita GDP-PPP (purchase power parity) as an index of urban income. The intersection of the *x*-axis and *y*-axis represents the average per capita carbon inflow and per capita GDP-PPP of the cities. The impacts of the share of services sector (size scale) and population density (color scale) are also shown in the figure.

Figure 1 shows per capita total carbon inflow (TCI) and per capita gross domestic product in purchasing power parity (GDP-PPP) of 16 global cities. Here, TCI refers to the sum of physical carbon inputs to a city and fossil fuel combustion-related virtual carbon from upstream supply chains. TCI integrates two complementary accounts, physical carbon and fossil fuel-derived gaseous (virtual) carbon, and quantifies the scale of a city’s carbon metabolism. We use carbon (C) as the unit of TCI for consistency in that it includes flows of physical carbon content in products (in C) as well as fossil fuel combustion-related virtual carbon emissions (CO_2_, which is then converted to C).

In the figure, four quadrants are separated based on the magnitude of per capita carbon inflow and urban income, showing groups of cities with high-income and high-carbon inflows (Q1), low-income but high carbon inflows (Q2), low-income and low-carbon inflows (Q3), and finally, high-income but low-carbon inflows (Q4). These are relative situations applied within this sample of cities, with quadrants separated by the average value of per capita TCI and per capita GDP-PPP of these 16 cities. We find that per capita TCI of Singapore is the highest (12.0 t C) in the study cities, which is over 4 times of that in Sao Paulo (2.7 t C). By and large, TCI is positively correlated with urban income (represented by per capita GDP-PPP), though a higher per capita urban income does not necessarily result in a larger per capita carbon inflow. For example, Moscow and Hong Kong in Q2 have a relatively high level of carbon inflow and a lower urban income than other cities located in Q1 (such as Los Angeles and New York). In comparison, Vienna and London in Q4 are able to have a relatively low per capita carbon inflow and at the same time, a high urban income. Cities in Q3, in this study, are mainly located in developing countries, which have a per capita carbon inflow less than 5.0 t C to their economies, notably smaller than the high-income group of cities in Q1 which have a per capita carbon inflow of over 6.9 t C.

A higher income may signify a bigger expenditure on products and services purchased from local or external markets, but evidently, the carbon inflow to a city can also be influenced by other socioeconomic factors such as economic structure and urban form, other than affluence. There is no single factor that controls the diversity in the human appropriation of carbon in cities, albeit it can be partially explained by the increasing effect of the share of services sector and the decreasing effect of population density, as identified by the regressions (Supplementary Fig. 1). For instance, within the income range of $38,600–$42,000/year and the population density range of 4200–6800 inh km^−2^, per capita TCI of Stockholm is only 58% of that in Singapore, partially explained by the higher proportion of services (and less manufacturing) in the former city. In contrast, with an approximate urban income (around $40,500/year) and a similar share of services (65%), Toronto is reported to have a bigger per capita TCI than Tokyo, partially because the former has a lower urban population density, which is relevant to shorter commuting distance and more energy-efficient infrastructure, such as district heating systems and public transportation. As counter-examples, Beijing has the second lowest TCI/capita, although manufacturing (such as power plants inside the city) is still an important sector for the city in the investigated year and the share of services in its economy is much lower than other cities like London and Sydney. This requires further explanation from the contributions of physical and virtual flows as well as the varitions in cities’ sectoral structures.

**Fig. 2:**
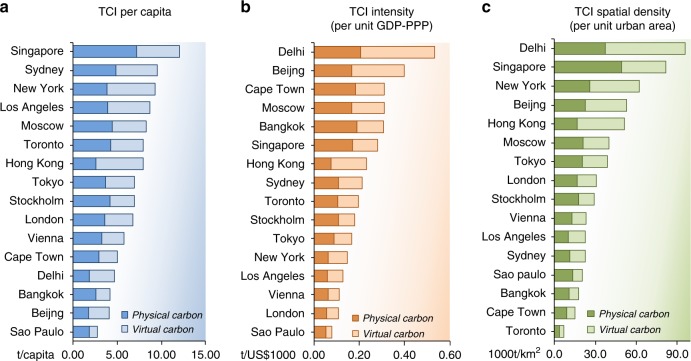
Contributions of physical and fossil fuel combustion-related virtual carbon to the total carbon inflow of global cities represented by (a) per capita (TCI per capita), (b) per GDP-PPP (TCI intensity), and (c) per urban area (TCI spatial density). The TCIs for the 16 global cities encompassing physical carbon and virtual carbon are shown by using various indicators. The contributions of physical and virtual carbon associated with urban economies vary greatly, resulting in different carbon performances of cities (represented by per capita inflow, intensity, and spatial density).

The variation in the TCIs of cities is explained by the different contributions of physical carbon as well as fossil-fuel derived virtual carbon associated with the urban economy (Fig. 2). We find that from 32 to 67% of the total carbon appropriated by cities is obtained from physical flows in products. The difference in physical carbon inflow explains up to half of the difference of per capita TCI between two cities. Manufacturing, supply of energy (such as electricity and gas) and construction sectors play an important role in physical carbon consumption given their high demands of fossil fuels (Supplementary Fig. 2). For example, the physical carbon inflow to Moscow (4.4 t C/capita) is mainly contributed by the big manufacturing and construction sectors in its economy. Transportation contributes more to the physical carbon in low-population-density cities such as Toronto and Los Angeles than in compact cities like Tokyo. A national-scale study^36^ estimated that the global average physical carbon was around 1.2 t C/capita in 2004. Our study finds the average physical carbon inflow of the 16 cities (3.5 t C/capita) nearly triples this global estimate, albeit differences in study boundaries, year of inventory and other aspects noted in Methods.

Fossil fuel combustion-related virtual carbon, as part of cities’ carbon metabolism via upstream activities, is also found to play a large part in TCI. Most cities in our study, outsource a considerable proportion of their carbon emissions by producing electricity upstream and importing materials, goods and services, significantly amplifying the climatic impact of the urban economy, similar to observations in prior studies^14,19,20^. Peters et al.^36^ reported virtual gaseous carbon contributed half of total carbon related to international trade. Here, we find a similar share of virtual carbon in the TCI of urban economies. These results manifest that, if upstream activities are excluded, the urban impact on the carbon metabolism will be highly underestimated. Nevertheless, the share of virtual emissions among cities is ranging widely from 33% in Sao Paulo to 68% in Hong Kong. The small virtual carbon emission embodied in goods and services purchased by a citizen in Beijing on average (2.4 t C/capita) largely explains why this city, albeit having many manufacturing industries and power plants within its boundary in the study year, has a relatively low per capita carbon inflow.

In addition to per capita inflow (Fig. 2a), the TCIs of cities are also compared based on per GDP-PPP (Fig. 2b) and per unit of urban area (Fig. 2c). The TCI intensity (meaning carbon inflow per unit GDP-PPP) of the cities varied notably, and ranged from 0.1 to 0.5 t C/$1000. The TCI intensity is high in Delhi, Beijing, and Cape Town, more than 4 times of the city with the lowest intensity (i.e., Sao Paulo). Of the 16 cities, TCI intensity is the highest in Delhi, mainly because almost all sectors of the city used carbon-intensive electricity (either gas- or coal-based power generation). Next to Delhi, cities like Beijing, Cape Town and Bangkok also have a high TCI intensity, mainly because of their material-intensive economies, power plants within boundaries and roles in global supply chains reliant on industrial production with relatively low value added and high energy intensity. In contrast, London and Vienna have a lower TCI intensity with a higher share of services in their urban economies. Our results show that the TCI spatial density (meaning carbon inflow per urban area) is the highest in Delhi (~96,000 t C km^−2^), followed by Singapore and New York, and is the lowest in Toronto (~7000 t C km^−2^). The difference of TCI density among cities is impacted by both the magnitude of imported carbon for their urban metabolism and the density of housing and public infrastructure. It should be noted that these results may be subject to considerable uncertainty from different sources, as described in the Methods.

### Physical carbon balances of cities

**Fig. 3:**
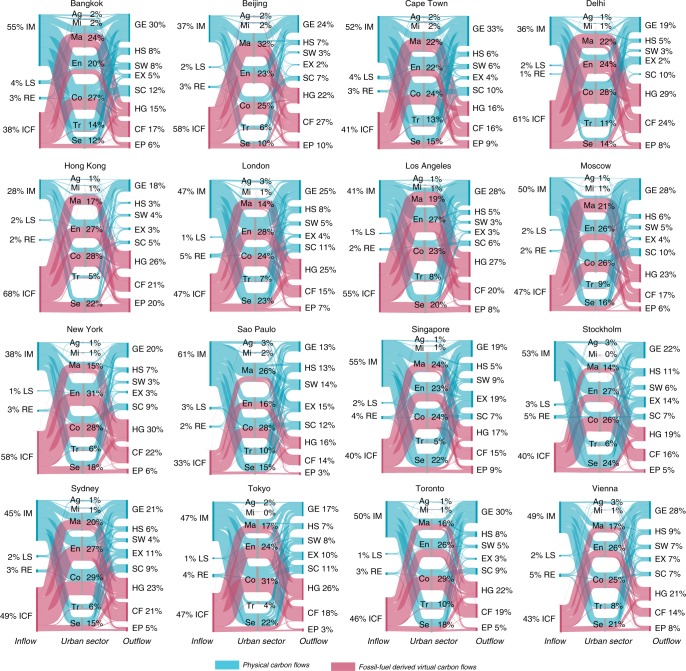
Physical carbon and fossil fuel-derived gaseous virtual carbon flows (excluding CH_4_) modeled for 16 global cities. These Sankey diagrams show the in- and outflows of physical carbon (in blue) and fossil fuel-derived virtual carbon (in red) associated with urban economic sectors. The numbers represent the proportions of flows to the total carbon balance of the respective city. The physical carbon inflows include: imports from other regions (IM), local supply by urban ecosystems (LS), and recycling of materials (RE), and physical carbon stocks and outflows, including household storage (HS), changes in carbon stock in urban sectors (SC), gaseous emissions (GE), solid waste (SW), and physcial export of carbon in goods (EX). Fossil fuel-derived virtual carbon embodied in import (ICF) to cities is accounted for, and is then allocated to flows driven by household and government expenditure (HG), fixed capital formation (CF), and exports as final demands (EP). Fossil-fuel derived virtual carbon flows are modeled using input–output analysis. The sectors are agriculture (Ag), mining (Mi), manufacturing (Ma), supply of energy (En), construction (Co), transportation (Tr), and services (Se).

Using the proposed framework, we quantitatively track the physical carbon flows of the 16 global cities from sources to economic sectors and then to change in stocks or outflows (Fig. 3). Most of the physical carbon manipulated by cities is obtained from imports (IM). In the study cities, between 88 and 92% of the physical carbon is gained from outside the urban boundary, while only between 2 and 6% is extracted from urban ecosystems, and between 3 and 8% is recovered from recycling (RE) of materials. The physical carbon import captures a very important part of the total carbon metabolism of the cities, whose contribution is 47% on average. All the cities rely heavily on external markets (domestic or global markets) to derive the physical carbon that supplies their urban economies. For cities, such as Moscow, Bangkok, and Cape Town, carbon imported in products accounts for more than half of their total carbon balances, while local extraction and recycling only contribute a very small fraction. The annual recycling of carbon content in Stockholm, Vienna, and Tokyo contributes around 8% of the input of physcial carbon (but <5% in terms of total carbon), still small compared with physcial carbon imports. Research has shown that much of the carbon emissions associated with consumption in urban areas are outsourced via global supply chains, and frequently to less-developed areas^15,16^. Here, we find that a dominant part of physical carbon used in urban production and consumption is also outsourced. This could amplify the already unequal exchange of gaseous emissions in trades, and considerably increase the complexity of managing carbon flows across boundaries. Supply of energy and construction of bulidings and infrastructure present a challenge to achieving low-carbon economies for cities like Beijing, Bangkok, and Cape Town, as they account for nearly half of the physical carbon inflow (Supplementary Fig. 2). In cities like London and Hong Kong service sectors should receive more attention, as they represent up to 25% of total physical carbon inflow.

The physical carbon inputs to cities have different metabolic fates, ending up as gaseous emissions (GE), solid waste (SW), household storage (HS), changes in stocks of urban economic sectors (SC), and physcial export in goods (EX). On average, a considerable amount of the carbon appropriated by cities immediately become GE (i.e. CO_2_) from combustion of fossil fuels by urban energy users. GE are ranging from 13 to 33% of the total carbon appropriated (23%, on average, or 1.6 t C/capita), with cities like Toronto, Moscow, and Los Angeles at the higher end of the spectrum. The energy supply sector dominated in many cities such as Hong Kong and New York accounting for about 40% of their total emissions, while transportation represented around 35% of the CO_2_ emission from Sao Paulo and Delhi.

The carbon stored in households as durable products (such as wooden furniture, textile, plastics, rubber, papers, and paperboard, but excluding fuels for cooking and driving) amounts to between 3 and 13% (or 0.2–0.8 t C/capita) of cities’ total carbon. Much of the difference in this household carbon results from the contribution of services reflecting the diverse demand levels and lifestyles between cities. Residents in cities like Moscow, Los Angeles, and Toronto store more carbon than people living in Delhi and Sao Paulo. This proportion of carbon is accumulated in durable goods purchased by urban households rather than immediately discarded or treated as waste. The difference is mainly in the speed of carbon released to the atmosphere depending on how long carbon is stored in households. In addition to household storage, a considerable part of physical carbon goes into industrial sectors and becomes part of the stock, which is between 5 and 12% of the total carbon balance (or 0.3–0.9 t C/capita). The construction sector makes a large contribution to stocks, and can be a huge component in rapidly expanding cities such as Beijing, Delhi, and Sao Paulo. Adding household storage and other urban stocks together, we find 8–24% (or 0.6–1.5 t C/capita) of the total carbon appropriated by cities is stored within the year of investigation, which is comparable to the carbon already emitted to the atmosphere for energy use in many cities investigated here. The carbon stored in the form of durable products, infrastructure, buildings, or production facilities can be emitted after a time delay, depending on the nature of the stock. This stored carbon in cities is found to be over two times of the global average per capita physical carbon stock (in wooden and petroleum products) estimated by Peters et al.^36^. Albeit with a different system boundary and research scale from their study, these results may indicate that lives and production in cities accumulate much more carbon than the rest of the global economy on a per capita level. Similar findings can also be found in energy and material flows concentrated in cities (e.g., ^41,42^).

The exports of physical carbon from cities are much smaller than their imports. Around 6% (or 0.5 t C/capita) of the total carbon appropriated by cities is exported or re-exported to other regions as products. A large amount of this exported carbon is from the service sectors (e.g., wholesale and retail trade) and is large in cities like Singapore and Hong Kong. In addition, another 6% (or 0.4 t C/capita on average) of the total carbon becomes solid waste that may be disposed off within or outside the urban boundary. Given current treatment technologies, this carbon is rarely recycled back to the urban economy, and may have been partially released into the atmosphere through waste treatment processes such as incineration.

Carbon sequestration by urban trees is found to be small in this study compared to the total urban carbon metabolism (Supplementary Fig. 3). Urban carbon sequestration only offsets, on average, 2% of the territorial carbon emissions from cities and less than 1% of their TCI, albeit with considerable uncertainty introduced by estimating forest land cover and selecting indicators of sequestration. Even for cities like Sao Paulo and Bangkok, where urban forests occupy large areas of land, the possibilities for offsetting through natural sinks are limited. The low rates of carbon sequestration by trees in cities were also reported in other studies^27,28^. While trees in cities have benefits for improving air quality and regulating microclimates, it may be a more realistic option to create natural sinks outside urban boundaries where the opportunity costs would be lower.

### Virtual carbon balances of cities

We also show the fossil fuel combustion-related virtual carbon of cities and how it is attributed to urban sectors in terms of different final demand categories (household and government consumption, capital formation, and export) (Fig. 3). Studies have reported that upstream emissons have a considerable influence on the urban carbon balance^14,15,20^. Our work further articulates that upstream emissions are significant even when they are accounted for in a broader context of the carbon metabolism that includes both physical and virtual carbon streams. We find that in the whole balance, 30–53% of total carbon is driven by local consumption and investment (i.e., household and government consumption and capital formation) of the cities as gaseous upstream emissions (2.8 t C/capita, on average), while gaseous carbon embodied in export as a final demand contributes a smaller part, varying from 3 to 20% of the total balance (0.5 t C/capita, on average).

There is a high diversity in the fossil fuel combustion-related virtual carbon driven by urban demands. Cities with high income tend to have a bigger share of virtual emissions driven by household and government consumption (Supplementary Fig. 4). The supply of energy and services sector make a large contribution to the virtual carbon emissions of many cities (Supplementary Fig. 5). For example, household and government consumption in Tokyo, New York and Los Angeles accounts for more than 30% of their total carbon balances due to their large imports of electricity for local consumption and products for services sector, and this is over half of total virtual carbon emissions associated with these cities. In comparison, higher proportions of the virtual emissions are driven by capital formation and export in Beijing, Bangkok, Delhi, and Sao Paulo. For example, 45% of Beijing’s virtual emissions are associated with capital formation because of construction of new buildings and infrastructure as well as purchase of industrial equipment.

## Discussion

Cities, important stores of carbon, play an important part in the global carbon balance and in tackling climate change^3,4,25^. Current frameworks for city-level carbon accounting were mainly developed to capture gaseous carbon emissions within or across boundaries (e.g., ^12,15,23^). They concentrate on determining how much gaseous emission can be attributed to urban activities and provide a basis for adopting emissions reduction targets based on historical and current emission trajectories. In relation to these efforts, the quantification and modeling of both physical carbon and virtual carbon associated with an urban economy could be a new and complementary perspective for decarbonization. Based on an integrated framework, we can target both what has been emitted into the atmosphere and what may come in the future that will influence climate change mitigation. The inputs, distribution, and metabolic fate of carbon appropriated by cities can be tracked based on a harmonized global urban dataset. A meaningful comparison between different streams of carbon, such as carbon imports and exports, or carbon that is transformed into stocks versus gaseous carbon can be made within this consistent framework.

The proposed indicators could provide new insights into the carbon impacts of urban areas. The TCIs to global cities vary widely, regardless of whether they are measured in per capita, intensity, or spatial density units. Cities such as Vienna, London, and Tokyo exhibit a comparatively low-carbon pathway considering the whole supply chain. However, their approach may not be appropriate for all cities. While it may be, as suggested, possible for cities to form partnerships or collaborative networks (e.g., ^43,44^) when building a low-carbon future, it is difficult or even counterproductive to have the same one-size-fits-all mitigation approach. Scholars have recognized the construction of low-carbon roadmap for cities should not only be based on their existing emissions, but also on socioeconomic profile, infrastructure and other metabolic characteristics^41,42,45^, all factors that are important in restraining future carbon budgets shared by urban economies. But there are no simple correlations between single urban characteristics (such as population density, share of services sector and income) and the carbon impact, as found by our study and other research^42,45^. While it is still important to analyze the drivers underlying carbon emissions, an equally useful and straightforward cut-in point could be scrutinizing carbon flows in the entire urban metabolism, including physical and virtual carbon, and not just gaseous emissions. Ideally, cities’ low-carbon roadmaps should be based upon carbon flows at the urban sector level (or even at the process level) and from a life-cycle perspective, with their linkages to different metabolic pathways quantified and mapped.

Both physical carbon appropriated within the urban territory and virtual emissions occurring outside the urban boundaries strongly influence cities’ carbon metabolism, largely consistent with what has been found at national scale^36^. Although nearly half of the carbon metabolism is outsourced through production outside urban boundaries, a large amount of carbon trapped in imported products and stored in the economy can still be managed within the reach of the city. This stored carbon is found by this study to be at least twice as big as the global average per capita, but it is much less studied, especially at city level, than the existing carbon emissions impacting the climate. The carbon temporarily stored in households and other urban stocks can contribute a big potential for mitigation that is comparable to the amount of annual carbon emissions from within cities. In almost all study cities, household storage is found to be a significant carbon stock across different levels of income and stages of development, mostly because these carbon-containing products are essential for all societies (for housing, transport and other important aspects of living). Most of this household-related carbon will eventually be released into the atmosphere^25^, albeit this may take from a few years to many decades. Durable products and materials stored in households and other sectors (such as wooden furniture, textiles, plastics, and rubber) usually have a long service life and may undergo another long period of slow release to the atmosphere after usage. Therefore, cities can still take advantage of this time lag to manage, or at least at this point, regularly monitor their carbon inflows and carbon stocks. The so-called asymmetrical effect of changes and lags dependent on economic scale and carbon emission dynamics has been discussed in the literature^46^. Taking the carbon retained in urban durable goods into account, this asymmetrical effect could be larger and longer lasting than expected. This is because stock-originated emissions may continue to be released with some inertia even when economic growth has slowed down or stopped altogether.

Studies have indicated that committed carbon emissions from current urban infrastructure account for a big share of future GHGs^47,48^. In addition to the impact from buildings and updating infrastructure in cities, the less-studied aspect, i.e., the carbon trapped in these infrastruture and other durable products has also shown to be important as potential sources of future emissions. Investments in urban stocks (e.g., housing stock, production facilities, and infrastructure) have strong implications for carbon emissions during their lifetime. These stocks should also be regularly examined as part of the committed responsibility to decarbonization, and for the nonrecyclable stocks, timely action should be taken when they are disposed of as waste. Considerable evidence suggests a large amount of carbon is being emitted globally caused by solid waste disposed in landfills or to incineration^33,49,50^. In most regions and cities, there is no sorting system to separate carbon-containing waste (woods, plastics, etc.) from other waste before they are incinerated^32^. Therefore, a significant potential for climate change mitigation lies in managing the urban stocks during their lifetime. Taking the modeling of carbon metabolism of cities as a first step, more research is needed to track urban durable products and their potential fates as emissions from landfill, combustion (either on open lands or in waste-to-fuel plants), and reuse or recycling. Such research is critical for pinpointing which stock management options could be promising for cities in order to stabilize future global climate.

## Methods

### Relationship to previous studies

In this study, an integrated approach is developed to track various metabolic flows of physical carbon through cities as well as flows of fossil fuel combustion-related virtual carbon emissions attributed to urban demands. It provides a different perspective from the inventories of gaseous carbon emissions based on energy consumption and industrial processes (e.g., citywide inventories^7,8^ based on IPCC guidelines^51^) as well as the modeling of embodied emissions in trade (e.g., ^15,19,20^). Our approach not only captures the historical carbon emissions that have already been released to the atmosphere (either from within or outside urban boundaries), but also identifies future potential emissions hidden in carbon flows such as changes in stock of households and other economic sectors. This latter point also distinguishes our framework from other carbon accounting schemes, such as the 3-scope Greenhouse Gas Protocol proposed by ICLEI, WRI, and C40^11,12^, and the wide production-^19^, infrastructure-,^15^ and consumption-based carbon footprints^17^ that concentrate their accounts on fossil-fuel related GHG emissions and target economic activities causing these emissions.

There has been research that linked the urban metabolic framework to footprint analysis by combing MFA with life-cycle approaches (such as LCA and IOA) and evaluated the environmental impacts of cities, for example, the works of Hillman and Ramaswami^21^, Goldstein et al.^52^, as well as other related models at country level (e.g., Eco-LCA nitrogen model for the US economy^53^ and MFA-IOA material footprint model for nations^37^). A national-scale study by Peters et al.^36^ synthesized CO_2_ embodied in import as well as physical carbon present in materials, with a focus of quantifying total carbon linked by international trade. The integration of MFA, LCA, and IOA here has a different focus from the abovementioned studies, i.e., consistently tracking various metabolic pathways of physical carbon from inflows to emissions, changes in urban stocks, solid waste or physcial export as well as virtual emission from import to final demand categories. Hao et al.^54^ quantified several types of urban carbon flows and stocks for one mountainous Chinese city at a very aggregate level, different from the detailed sector-based accounting and modeling of various metabolic pathways conducted in this study, where a harmonized carbon dataset of global cities is compiled and used. We also illustrate the linkages to and differences from previous studies in Supplementary Fig. 6.

### Integrated framework for carbon metabolism

**Fig. 4:**
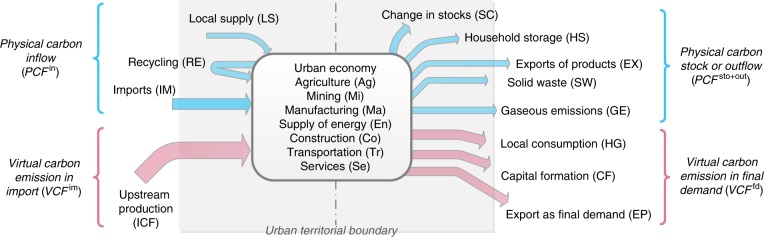
Framework for tracking urban physical and virtual carbon metabolism. To capture the broader carbon impact of an urban economy, we combine physical carbon account and fossil fuel-derived virtual carbon account within a consistent framework of carbon metabolism. First, we track the physical carbon appropriated by a city as goods or raw materials imported from outside (IM), local supply from urban ecosystems (LS), or recycling of materials (RE), and how this carbon is distributed within the urban economy and become part of household storage (HS), changes in stock industrial sectors (SC), gaseous emissions (GE), solid waste (SW), or physical exports as goods (EX). Second, we model the virtual carbon emissions manipulated by a city through its import of products and further allocate them to local (household and government) consumption (HG), capital formation (CF), and exports as final demand (EP).

The technical framework for modeling the physical carbon and the fossil fuel combustion-related virtual carbon metabolism in cities is illustrated in Fig. 4. All the carbon flows of the urban economy are tracked and allocated to seven aggregate economic sectors: agriculture (Ag); mining (Mi); manufacturing (Ma); supply of electricity, gas, and hot water (En); construction (Co); transportation (Tr), and services (Se). This integrated framework allows us to trace the inflows, stocks, and outflows of physical carbon content and virtual emissions associated with these urban sectors. Currently, city-scale carbon inventories are mainly based on energy flow analyses, life-cycle analyses, or hybrid models that track emissions precede urban consumption (e.g., ^20–22^). By integrating MFA, LCA, and IOA, our approach encompasses both flows of physical carbon and fossil fuel combustion-related virtual carbon, which could provide a broader view on the carbon impact of urban activities and which flows to target for a more systemic and informed carbon emission mitigation.

### Scope and consistency

The flows of physical carbon in the paper are quantified based on the carbon content of products and materials, consistent with the definition in existing urban carbon inventories^25,29^ and national flow inventories^30,36^. In addition, the flows of fossil fuel combustion-related virtual carbon are modeled based on upstream carbon emission (CO_2_), excluding the upstream carbon content in products that is indirectly linked to the carbon inflow, consistent with the definition of virtual carbon commonly used in the majority of literature (e.g., ^38,39^). For example, in terms of food, we consider the carbon content in food products as well as upstream gaseous virtual carbon for food production and processing, but exclude other upstream non-gaseous carbon (e.g., the carbon content of fertilizers used in agricultural production). The same can be extended to the scopes of other physical carbon flows. Accounting for the upstream physical carbon content based on direct urban carbon consumption data and city-level IO table, at this stage, is difficult since some of the physical flows during the extraction and processing (such as the loss of carbon during cropping, harvesting, and processing) will not be captured^55^. Current material extraction datasets are mostly developed at the national level^37^ and no worldwide data exist for cities. We focus our global-city study on the physical carbon in products inflows to and fossil fuel combustion-related virtual carbon with a clearly-defined boundary that matches with city-scale metabolic data. Despite the exclusion of the upstream carbon content, the carbon inventory in the model is self-consistent in that the physical carbon balance from inflows to outflows is accounted for independently from the balance of virtual emissions. The city-level energy and materials data compiled meet our research goal of tracking carbon metabolic pathways through cities.

### Linkage of the metabolic framework to climate policy

In this study, we do not intend to quantify exactly how much GHG will be emitted from each type of carbon-containing products. Instead, we aim to unlock the potential of carbon mitigation hidden in the less-studied carbon flows attributed to cities. We differentiate what have already become gaseous emissions and what have not yet become emissions (such as urban stocks) but still hold the potential of releasing carbon into the atmosphere over their remaining life cycles. Most urban stocks will eventually end up in waste after the service life of products^25^. Studies have quantified the GHG emissions from a range of techniques applied in solid waste disposal. The World Bank reported over 90% of waste is burned or dumped on roads, open land, or waterways in many low-income countries^32^, contributing to a large amount of unintended CO_2_ or CH_4_ emissions^33,49^. Cities transport part of the waste outside their boundaries for landfilling, and in this process a considerable amount of unaccounted carbon emissions leak into the atmosphere. Incineration (e.g., using waste for electricity generation or heating) is frequently used for waste disposal, especially in high-income regions. Evidence shows that from a life-cycle perspective, incineration will still cause net GHG emissions even when solid waste is incinerated in modern facilities compared to recycling of these materials (e.g., ^33,56,57^). The strong implication of solid waste for climate change has also been acknowledged by global-scale studies, such as analyses on plastic waste^50^ and food waste^58^. There have already been discussions of including stock management in climate change mitigation (e.g., ^34–36^). In relevance to these efforts, we portray the carbon metabolic pathways through cities and identify the potential of decarbonization from managing urban stocks.

### Accounting of physical carbon flows

The accounting scheme for physical carbon is guided by standards established in MFA (e.g., ^37,59^). They are adapted to city-scale inventory of physical carbon from inflows to stock changes and to outflows at a detailed sector level. Physical carbon inflows (PCF^in^) account for the carbon content in goods and raw materials imported to the urban economy (IM, see Supplementary Table 1); recycling of carbon content in materials within the urban economy (through a city’s refuse reclamation); and local supply of carbon from urban ecosystems (local supplies (LS), such as biomass extracted from urban forests and parks). At the other end, the physical carbon stock and outflows (PCF^sto+out^) are represented by five flow categories (or metabolic outputs): household storage (HS), changes in carbon stock in industrial sectors (SC), gaseous emissions (GE), solid waste (SW), and physcial export (EX). Territorial carbon emissions mainly originate from imported fossil fuels and are released during energy uses inside the city. They are tracked and quantified based on IPCC guidelines for GHG inventory^51^. HS accounts for carbon that is stored in households for more than 1 year (such as wooden furniture and other durable products), while the carbon in less durable products such as food becomes part of SW. The change in the carbon stocks in all urban sectors (in the form of buildings, infrastructure, and capital goods) excluding household purchases are accounted for by SC. Finally, EX represents the carbon contents in products that are shipped to other regions.

Similar to prior carbon metabolic studies (e.g., ^26,29,34^), we convert key imported products and materials to their corresponding carbon content (i.e., the amount of carbon they contain). These products and materials, include food, fossil fuels (e.g. coal, coke, petroleum, and natural gas) for transportation, industrial and residential use, construction materials (wood, cement, and steel), carbon-intensive products (plastics, rubber, glass, and paper), furniture, and electronic goods. The physical carbon inflow $({\mathrm{PCF}}_i^{{\mathrm{in}}})$, and stock change and outflow $({\mathrm{PCF}}_i^{{\mathrm{sto + out}}})$ related to sector *i* of an urban economy are represented as follows

1 $${\mathrm{PCF}}_i^{{\mathrm{in}}} = {\mathrm{IM}}_i + {\mathrm{LS}}_i + {\mathrm{RE}}_i,$$

2 $${\mathrm{PCF}}_i^{{\mathrm{sto + out}}} = {\mathrm{HS}}_i + {\mathrm{GE}}_i + {\mathrm{SW}}_i + {\mathrm{EX}}_i + {\mathrm{SC}}_i,$$

where carbon is appropriated by the cities through IM, LS, and RE, and then allocated to several changes in stock and outflows, including HS, SC, GE, SW, and EX. The total of all physical inflows is equal to the annual changes in the carbon stock and outflows of the urban economy (including all *n* urban economic sectors). The physical carbon balance is established as

3 $$\mathop {\sum}\limits_{i = 1}^n {{\mathrm{PCF}}_i^{{\mathrm{in}}}} = \mathop {\sum}\limits_{i = 1}^n {{\mathrm{PCF}}n_i^{{\mathrm{sto + out}}}}.$$

Carbon sequestration by urban trees represents a natural carbon sink in urban areas. Through this process, the amount of CO_2_ released into the atmosphere can be reduced to varying degrees, depending on land use and vegetation distribution in the urban area. The flow of carbon sequestration is analyzed to articulate by how much urban ecosystems can offset emissions from cities in the context of urban metabolism. As in previous studies (e.g., ^27,28^), we use forest coverage and reference values of the carbon sequestration rate for each city to estimate the capacity for carbon sequestration by urban trees.

### Modeling of fossil fuel-derived virtual carbon flows

The second part of the urban carbon metabolism involves the tracking of fossil fuel combustion-related virtual carbon (VCF), including carbon emissions embodied in imports of electricity and other goods and services to a city. It is important to note that, when computing gaseous virtual carbon, carbon emissions from in-city energy use and industrial processes are excluded as they are already included in the physical carbon (as GE).

Many studies have used IOA to compute virtual (or upstream) carbon flows (e.g., ^17,19,20^). IOA is useful for tracking urban carbon flows since it captures the entire supply chains related to the urban economy. However, there are fewer IO tables complied for cities than for nations. The distinct economic characteristics of cities are only partly considered when national IO tables are downscaled and used to estimate the urban footprint. LCA provides an alternative approach for accounting for emissions from upstream and downstream processes without the constrains of IO table ^14,52^. In this study, a hybrid life-cycle analysis is used to model virtual carbon emission at an urban scale. First, we quantify the import-related carbon emissions and respective carbon intensities (i.e., carbon emissions per unit of output) based on LCA. These carbon intensities differentiate production technologies for supplying products to a city. For example, electricity-related carbon intensity is calculated from the different energy mixes in a city’s power grid. On this basis, we are able to quantify the virtual carbon embodied in upstream production for cities. The import-related virtual emission is further allocated to a city’s final demand categories (VCF^fd^). These final demand categories, consistent with mainstream IO models, include household and government consumption, capital formation, and exports.

4 $$k_i = {\mathrm{VCF}}_i^{{\mathrm{im}}}/X_i,$$

5 $${\mathrm{VCF}}_i^{{\mathrm{fd}}} = {\mathbf{k}}_i({\mathbf{I}} - {\mathbf{A}})^{ - 1}{\mathbf{y}}_i^{{\mathrm{HG}}} + {\mathbf{k}}_i({\mathbf{I}} - {\mathbf{A}})^{ - 1}{\mathbf{y}}_i^{{\mathrm{CF}}} + {\mathbf{k}}_i({\mathbf{I}} - {\mathbf{A}})^{ - 1}{\mathbf{y}}_i^{{\mathrm{EP}}},$$

6 $$\mathop {\sum}\limits_{i = 1}^n {{\mathrm{VCF}}_i^{{\mathrm{im}}}} = \mathop {\sum}\limits_{i = 1}^n {{\mathrm{VCF}}_i^{{\mathrm{fd}}}},$$

where ${\mathrm{VCF}}_i^{{\mathrm{im}}}$ represents the life-cycle carbon emissions embodied in upstream production of products imported to Sector *i*; *X*_*i*_ is the total output of sector *i*, ***y***_*i*_ is the urban final demand; *k*_*i*_ is the carbon intensity of import to sector *i*; (**I−A**)^*−1*^ is the Leontief inverse matrix, in which **A** represents the technical (input) coefficients of the urban economy, and **I** is the identity matrix; VCF_*i*_^fd^ represents the virtual emissions of sector *i* that can be allocated to different categories of urban final demand, they are local (household and government) consumption $({\mathbf{y}}_i^{{\mathrm{HG}}})$, capital formation $({\mathbf{y}}_i^{{\mathrm{CF}}})$, and exports $({\mathbf{y}}_i^{{\mathrm{EP}}})$.

Combing the streams of physical carbon and fossil fuel combustion-related virtual carbon, we derive the total carbon balance of the urban economy, or so-called total carbon inflow (TCI), as shown in Eq. (7). TCI can be used to represent the carbon impact of cities from a urban metabolc perspective. Different from the measurement of carbon footprint, it provides an alternative angle on how cities can improve their carbon performances considering flows related to current and future emissions. In this study, three indicators, per capita TCI (carbon inflow allocated to an urban citizen), TCI intensity (carbon inflow per GDP-PPP) and TCI density (carbon inflow per urban area) are proposed and used to compare cities’ carbon performances.

7 $${\rm{TCI}}{\mathrm{ = }}\mathop {\sum}\limits_{i = 1}^n {{\rm{PCF}}_i^{{\mathrm{in}}}} {\mathrm{ + }}\mathop {\sum}\limits_{i = 1}^n {{\rm{VCF}}_i^{{\mathrm{im}}}} {\mathrm{ = }}\mathop {\sum}\limits_{i = 1}^n {{\rm{PCF}}_i^{{\mathrm{sto + out}}}} {\mathrm{ + }}\mathop {\sum}\limits_{i = 1}^n {{\rm{VCF}}_i^{{\mathrm{fd}}}}.$$

### Data compilation

To ensure relevant urban activities are included, and to facilitate intercity comparison, the urban areas of the study cities are defined by local official urban statistics and are consistent with metabolic data. The comparisons are made around 2008 for consistency, when urban metabolic data are most available for the 16 cities, namely, Bangkok (2007), Beijing (2008), Hong Kong (2006), Delhi (2007), Tokyo (2008), Singapore (2007), Stockholm (2009), London (2005), Vienna (2005), Moscow (2009), New York (2009), Los Angeles (2008), Toronto (2007), Cape Town (2006), Sao Paulo (2009), and Sydney (2008). A description of these cities is provided in Supplementary Table 2. These cities are selected mainly because of their size, function and importance in the global economy, coverage of samples in different countries across major continents, as well as pragmatic reasons such as accessibility of urban metabolic data. Of course, this small number of cities is no way a representative of all cities but provides proof of concept as well as already some new insights into the carbon metabolism of cities.

Urban MFA data (such as material imports/exports and stocks in products, energy supplied and consumed, gaseous carbon emission and solid waste) are used to account for cities’ physical carbon. Energy and material flow databases are more accessible at national level. Using national databases, Peters et al.^36^ included fossil fuels, petroleum-derived products, harvested wood products, crops, and livestock products in their inventory of physical carbon, but excluded some other products such as household use of wood, paper and paperboard. As a city-scale study, we compile the physical carbon dataset from multiple sources (Supplementary Table 3): data obtained from records of the city or local researchers, data from publications/reports of the same boundary, and data extrapolated from the national- or regional-scale to the city-scale based on ratios. Waste data are mainly derived from city-scale environmental statistics. For example, the waste outflow and material recycling data of Delhi are derived from the Department of Environment of National Capital Territory of Delhi. Note that here we do not differentiate between solid waste treated inside and  outside the urban territory, as long as it comes from the economic sectors of a city. The carbon content factors are collected from various sources and compiled to match with our model (see the approach and references in Supplementary Note 1). Gaseous carbon emissions in this study only account for CO_2_ (in C), while CH_4_ and other GHG emissions are excluded.

IO tables for Beijing, Hong Kong, Singapore, London and Sydney are available from official statistics or published literature. The IO tables of New York and Los Angeles can be derived from IMPLAN^60^. For cities without such data (i.e., Bangkok, Delhi, Tokyo, Stockholm, Vienna, Moscow, Toronto, Cape Town, and Sao Paulo), city-level IO tables are downscaled from national tables based on location quotients (LQs) and cross-industry quotients (CIQs), a technique that has been widely used in applied economic studies^61^. The sectoral values added pertaining to these cities are used to represent city-specific LQs and CIQs (Supplementary Table 4), which have been frequently used as constraints in IO table compilation (e.g., ^62^). The IO tables simulated for these nine cities estimate their urban production structures and are able to link cities’ final demands to their carbon flows, albeit they could increase the uncertainty of the virtual carbon results. The table compilation process is described in Supplementary Note 2.

### Model uncertainty

Model results may have been influenced by a range of uncertainties that can only be partially quantified in this study. We are able to capture the portion of uncertainty in certain modeling processes (i.e., determination of carbon intensity and rebalancing of IO tables) based on a standard deviation approach that has been commonly used in environmental IO models (e.g., ^63^) (Supplementary Fig. 7 and Supplementary Note 3). These modeling processes may cause up to ±30% deviation of the virtual gaseous carbon across the 16 cities (as estimated in Supplementary Table 5). These results are subject to different types of uncertainty considering IO tables have rarely been matched to material flows, and there are other uncertainties on constructing the IO tables in disaggregating material flows to sectors in imports and exports that were reported in the literature^15,64^. These uncertainties are not known particularly in the 9 cities where IO tables are not available. In terms of physical flows, we estimate the uncertainty introduced by carbon content factors of products (up to ±26%, Supplementary Table 6), while other uncertain factors such as raw data consistency and sectoral disaggregation also need to be cautioned about, and ad hoc control techniques are usually helpful (Supplementary Table 7). For example, we use the citywide energy and material survey data (often in total) to constrain the sectoral decomposed metabolic data applied to a larger geographical scale.

### Limitations

First, metabolic data availability at the urban scale may limit the application of this approach. While energy and emission data are usually accessible, data about urban material metabolism are often scarce and may be subject to certain inconsistency because of a lack of common reporting standards. Fully consistent city-level metabolic database should be established globally to increase model accuracy. Also, establishment of a time-series carbon database will enable analyses of urban carbon dynamics in the future, such as fluctuations in carbon stocks that might occur when there are new investments in infrastructure and housing. Second, here, gaseous carbon emissions refer to CO_2_ rather than all GHGs. Although there could be CH_4_ emissions from farming, landfills and other activities (inside or outside urban boundaries), in this study, CO_2_ is accounted for as the dominant GHG, similar with the carbon inventories in some prior studies^31,65^. Third, carbonating cement possibly offsets 2% of the global annual total CO_2_ emitted from human activities over its life span^66^, which could be a long period of over 80 years or a century because cement carbonation is a very slow process^67,68^. Hence, the sequestrated carbon by cement is considered insignificant in such a short timeframe (i.e., a one-year research period in this study) compared to the total carbon metabolism. This is open for future studies based on city-scale observation over a much longer timeframe.

## Supplementary information

Supplementary Information

## Data Availability

The sources of energy and material flow data for the 16 global cities are provided in Supplementary Table 3. Other supporting urban socioeconomic and metabolic data have been linked to the literature or websites cited in the paper.

## Code availability

Programming code for carbon flow model is available from the corresponding author on request.
