# Supplementary material for: Physical and virtual carbon metabolism of global cities
Source: Nat Commun. 2020 Jan 10;11:182. doi: 10.1038/s41467-019-13757-3 (PMC6954253; doi:10.1038/s41467-019-13757-3)
Supplement: Supplementary file 1 — Supplementary Information [file 41467_2019_13757_MOESM1_ESM.pdf]

Supplementary Information for

Physical and virtual carbon metabolism of global cities

Chen et al.

Supplementary Figures

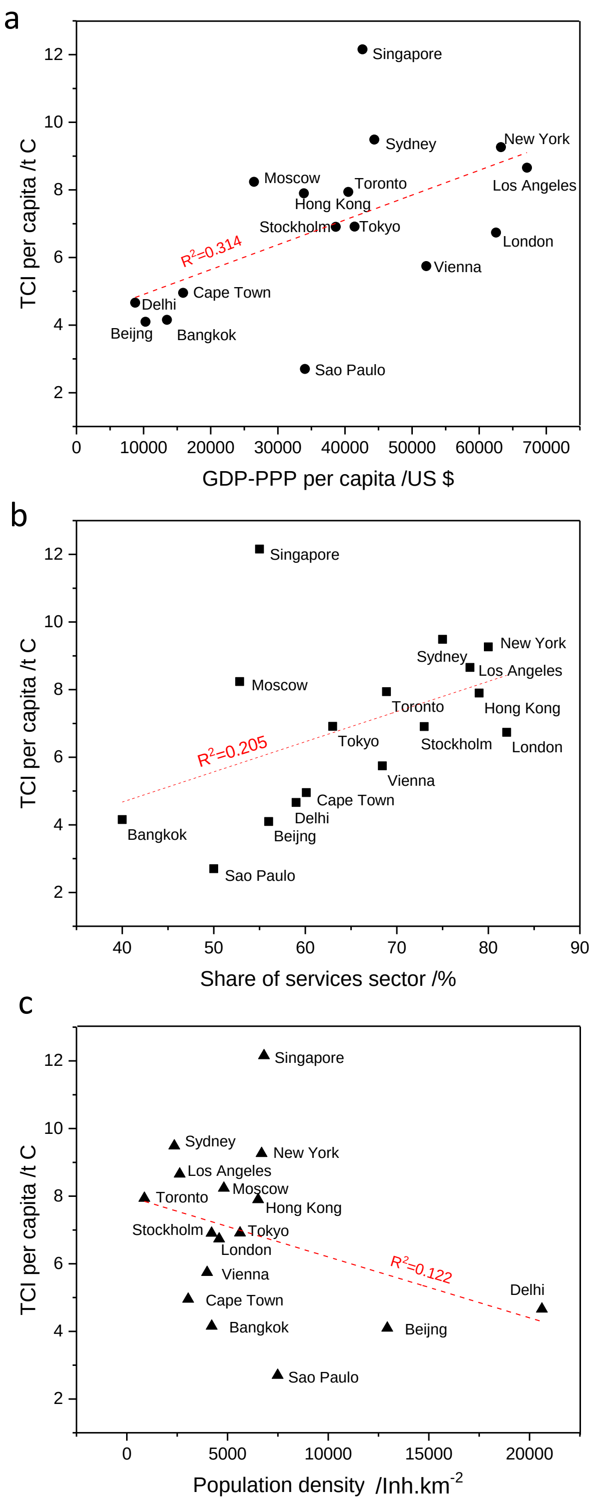

Supplementary Figure 1 Correlations of per capita TCI with (a) per capita GDP-PPP, (b) share of services sector and (c) population density

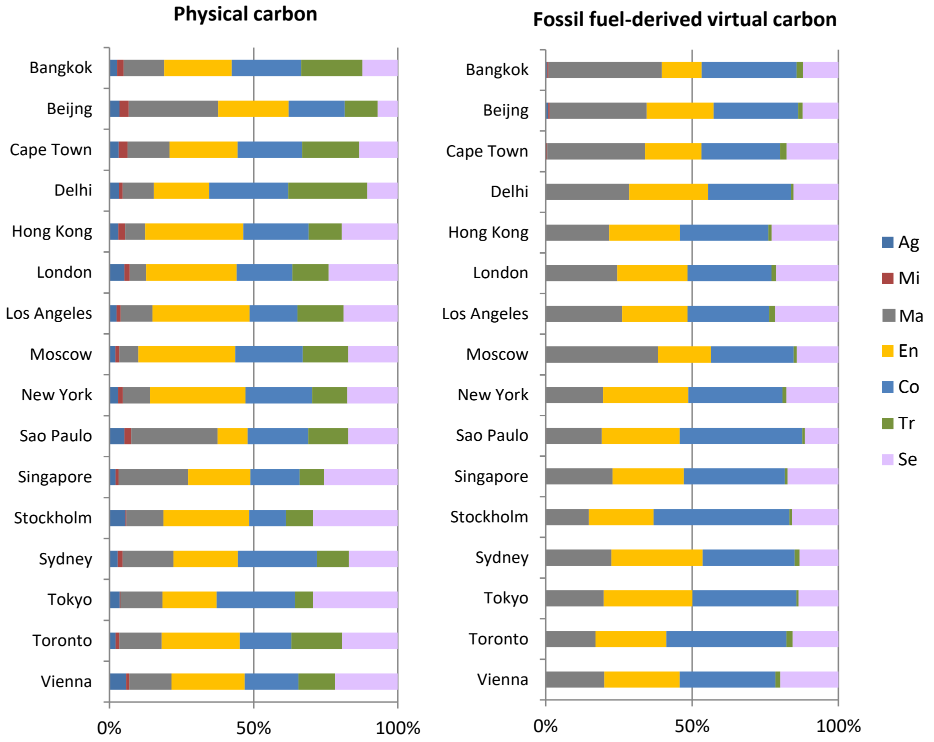

Supplementary Figure 2 Sector contribution to physical carbon flow (PCF) and fossil fuel-derived virtual carbon flow (VCF)

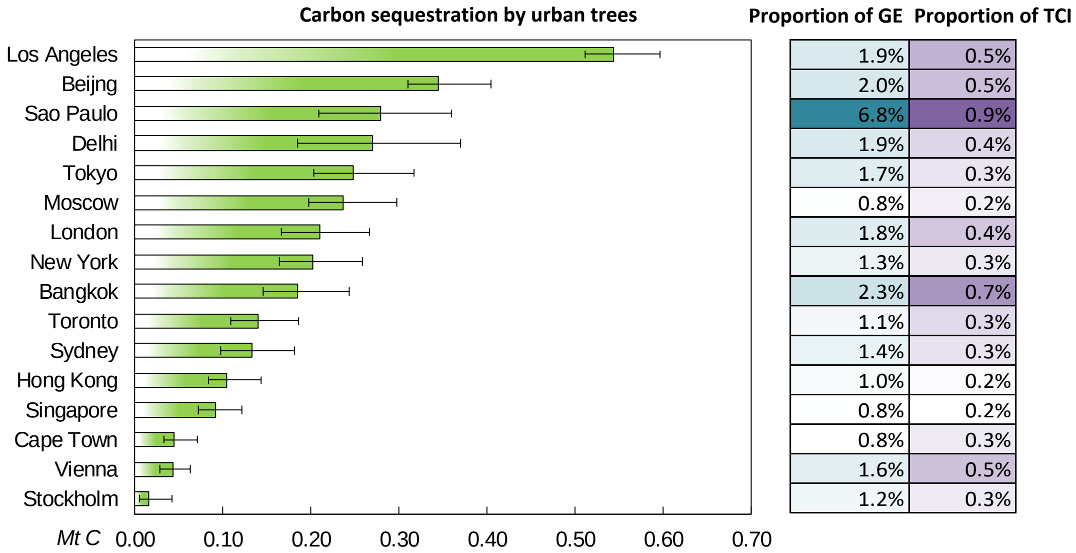

Supplementary Figure 3 Carbon sequestration by urban trees in cities

Note that when measuring the carbon sequestration in these urban areas, uncertainties (error bars in the figure) occur due to several factors (e.g. selection of sequestration parameters and data uncertainty of green coverage areas).

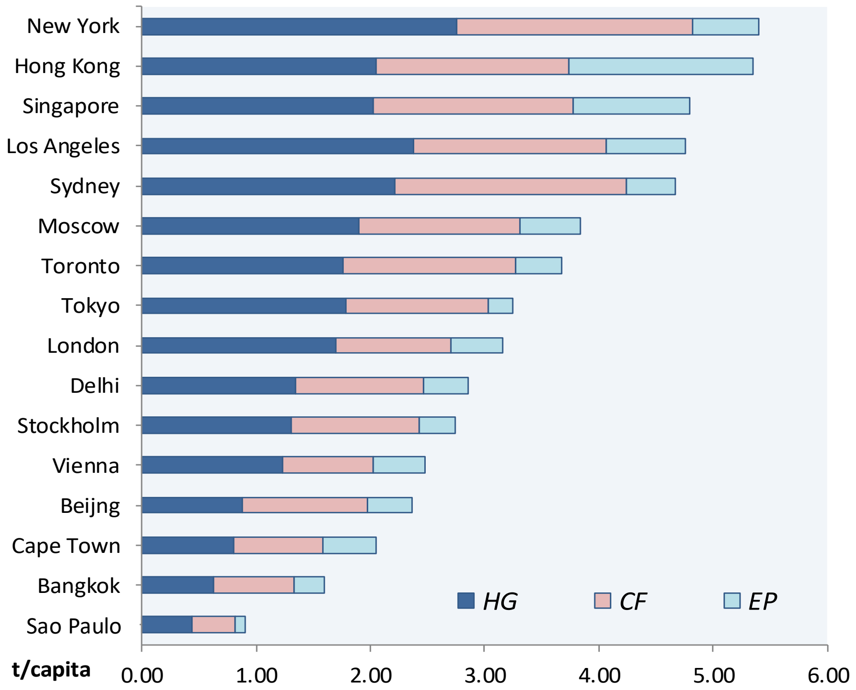

Supplementary Figure 4 Ranking of cities regarding virtual carbon emission driven by household and government consumption (HG), capital formation (CF) and export (EP) of cities

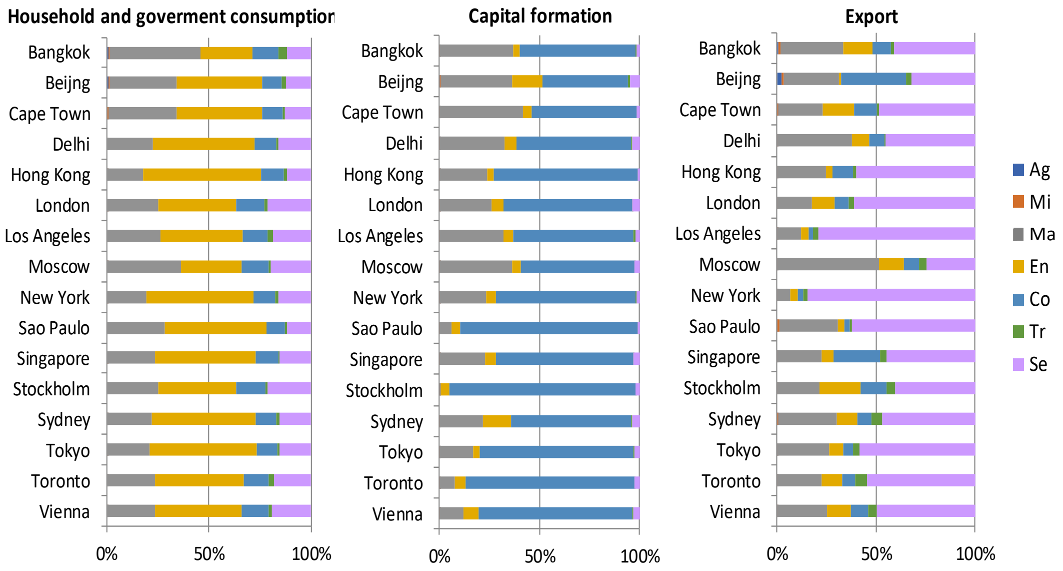

Supplementary Figure 5 Sector contribution to cities' virtual carbon flow driven by household and government consumption, capital formation and export

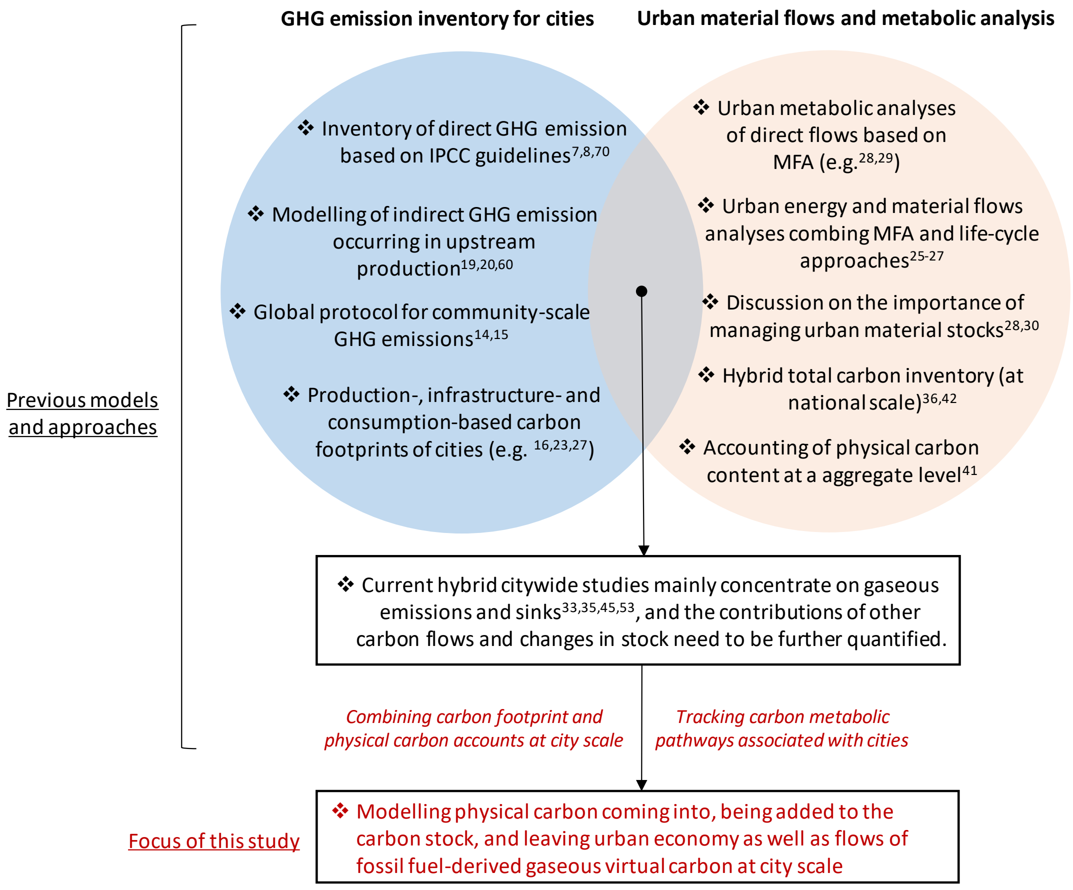

Supplementary Figure 6 Focus of this study compared to previous studies

Note that the cited references are relative to the References listed in the main text.

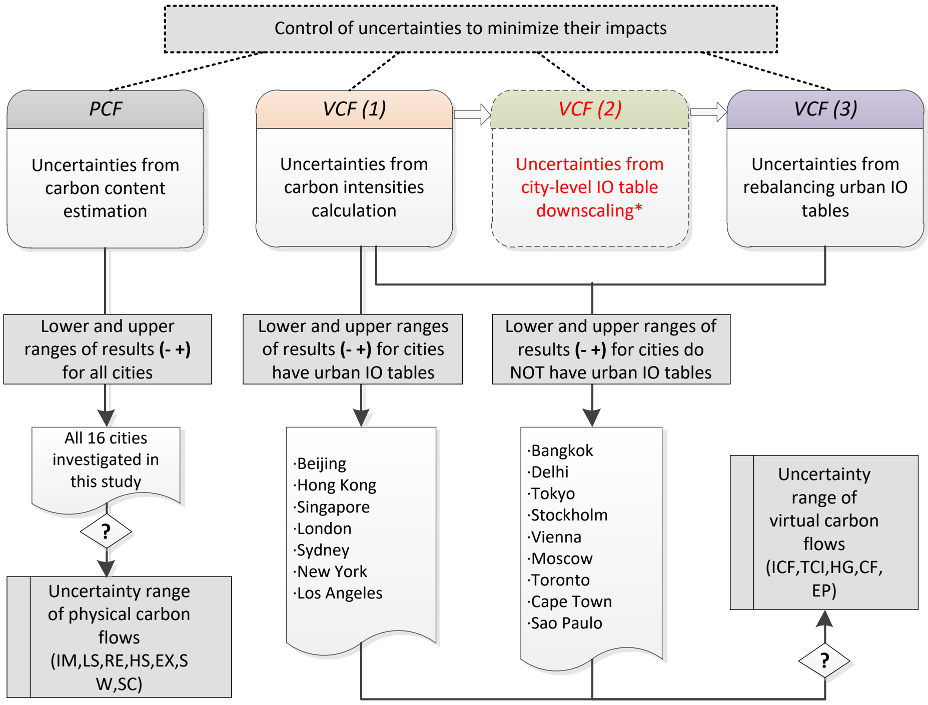

Supplementary Figure 7 Approach of uncertainty analysis in this study

*Note that the uncertainty analysis of the results for the 9 cities only consider the impacts of carbon intensity and rebalancing table (RAS), while the impact of downscaling national/regional IO table is only qualitatively discussed.

Supplementary Tables

Supplementary Table 1 A list of the main categories of product imported to cities

Categories of imported products	Sectors involved	Source/Explanation
Foods (grains, fruits, vegetable, meats, etc.)	Ag, Ma, Se	This category considered both finished foods and semi-manufactured foods, therefore Ag, Ma and Se were possibly involved with variation from city to city. For cities that have this data, we refer to the official reports and yearbook. For others, the city's import was broken down from national level based on United Nations Agricultural Survey for 2007[1] according to the proportion of population of the city to its country, thus making the simplifying assumption of equal per capita consumption of urban and rural populations. The emission factors data that do not exist in the software were referred to[2,3].
Construction materials (woods, cements)	Co	All the imports of concrete went to the construction sector and were redistributed to other sectors for different uses. The ratio of cement to concrete for Sao Paulo and Cape Town was assumed to be the same as that in Beijing.
Metals	Mi, Ma, Co, Se	Aluminum and steel were considered in this category. The life-cycle process covers the production and transportation based on the average distance between the source markets and the city.
Plastics, rubber, glass, papers, furniture	All respective sectors	Some of the data sources for this category are presented in Supplementary Table 4. The emission factors that do not exist in the software database were referred to a number of other references (such as plastic products[4], wooden furniture[5], paper[6])
Electronic goods	Ag, Ma, Se	The life-cycle processes for electronic goods imported to cities (e.g., batteries and computers) were referred to in references[7-9].
Transport	Tr	This category considered diesel and gasoline from cars and aviation (the data of Cape Town and Sao Paulo also considered motorcycles).
Electricity	En	All the imported electricity from external markets went to Po sector and was redistributed to other sectors for different uses. The emission factors considered different generation processes of power between cities. The recommended factors at national level were applied where those of cities were unavailable.
Thermal energy (coal, oils, gas)	All respective sectors	The data sources for this category are presented in Supplementary Table 4. The combined heat and power systems were calculated separately.

Supplementary Table 2. Demographic and economic attributes of 16 global cities

City	Location	Year	Population/million	Urban Area /km^2	Population density / inh. km^{-2}	GDP-PPP /billion US$	per capita GDP-PPP /US$	Share of services sector in economy
Bangkok	13N, 100E	2007	6.60	1565	4217	89	13485	40%
Beijing	39N, 116E	2008	17.70	1368	12939	182	10282	57%
Cape Town	33S, 18E	2006	3.46	1136	3046	55	15896	60%
Delhi	28N,77E	2007	16.12	782	20614	141	8747	59%
Hong Kong	22N, 114E	2006	7.20	1104	6522	244	33889	79%
London	51N, 0W	2005	7.20	1570	4586	450	62500	82%
Los Angeles	34N, 118W	2008	9.14	4494	2033	792	67119	78%
Moscow	55N,37E	2009	12.10	2510	4821	320	26446	53%
New York	43N, 75W	2009	8.12	1213	6694	513	63238	80%
Sao Paulo	23S,46W	2009	11.40	1522	7490	388	34035	50%
Singapore	1N, 103E	2007	4.88	716	6816	208	42623	55%
Stockholm	59N, 18E	2009	0.88	209	4211	34	38636	73%
Sydney	33S, 151E	2008	4.80	2036	2358	213	44375	75%
Tokyo	35N,135E	2008	12.30	2187	5624	510	41446	63%
Toronto	43N, 79W	2007	5.16	5905	874	209	40504	69%
Vienna	48N, 170W	2005	1.65	414	3986	86	52121	68%

Data sources: World Bank, United Nations, Global MetroMonitor and official statistics of cities

Supplementary Table 3 Data sources of carbon flows for 16 global cities

City	Type of data	Categories of data sources (by color) and special notes	Main references and sources
Bangkok, Thailand	Material imports/exports and stocks in products	Industrial material imports and exports are compiled based on 2010 data official reports and published studies on material flows of Bangkok.	Mehta et al.[10] National Statistical Office of Thailand[11] Department of Environment Bangkok Metropolitan Administration (BMA): https://iad.bangkok.go.th
	Energy supplied and consumed	Energy consumption data (primary and secondary) are verified with National Statistical Office of Thailand and Energy Policy and Planning office http://www.eppo.go.th.	Murakami et al.[12] National Statistical Office of Thailand[11] Phdungsilp[13,14]
	Gaseous carbon emission	Carbon emission is calculated by this study considering emission from energy and industrial activities. Compositions	https://www.seisakukikaku.metro.tokyo.jp/en/diplomacy/p

	and solid waste	and amount of solid waste are derived from Department of Environment of Bangkok Metropolitan Administration.	df/1011-05-shigen-e.pdf (Solid waste)
Beijing, China	Material imports/exports and stocks in products	Most of the data are acquired from local statistics, except that the stocks in urban households are adjusted from metropolitan area with its proportional GDP.	Campillo et al.[15] Beijing Municipal Bureau of Statistics[16] Editorial Board of China Commerce Yearbook[17] Editorial Board of China Iron and Steel Industry Yearbook[18] Editorial Board of China Mining Industry Yearbook[19] State Environmental Protection Administration of China[20]
	Energy supplied and consumed	The imported electricity and heat in the sectoral energy consumption table are reallocated to Energy sector in order to avoid double counting.	Beijing Municipal Bureau of Statistics[21] Beijing Statistics Bureau[22] Lu[23] Feng[24] http://www.bjstats.gov.cn/nj/main/2008-ch/index.htm http://tjj.beijing.gov.cn/nj/qxnj/2008/system/main.htm
	Gaseous carbon emission and solid waste	Carbon emission is calculated by this study considering emission from energy and industrial activities. Solid waste data is from China Environment Statistics.	China environmental statistics yearbook: http://www.stats.gov.cn/ztjc/ztsj/hjtjzl/2008/
Cape Town, SA	Material imports/exports and stocks in products	The material imports and household consumption are based on data in 2005; Export data are down-scaled from national level data according to the proportional industrial GDP of Cape Town in South Africa. This estimation constrained by the economic structure of Cape Town.	Department of Economic and Human Development, City of Cape Town[25,26] Campillo et al.[15]
	Energy supplied and consumed	Basic energy consumption data are from Statistics South Africa (http://www.statssa.gov.za) and World Energy Council website (https://www.worldenergy.org)	Swilling[27] Ward and Walsh[28]
	Gaseous carbon emission and solid waste	Solid waste data are from State of the Environment Report, City of Cape Town. Carbon emissions are extracted from C40 Cities Climate Leadership Group and matched with our sector categories.	City of Cape Town[29,30] C40 Cities Climate Leadership Group: https://www.c40.org/
Delhi, India	Material imports/exports and stocks in products	Construction materials only consider cement and woods. Energy fuels of sectors only consider fossil fuels. Recycling data was obtained from informal recycle sector (http://www.chintan-india.org/) that	Delhi Statistical Handbook[31] Ruet et al.[32] Streicher-Porte et al.[33]

		includes recycling of plastic and other carbon-bearing materials.	
	Energy supplied and consumed	Energy consumption and power generation data are from Energy Statistics Central Statistics Office of India.	Central Statistics Office of India, Ministry of Statistics and Programme[34] Implementation: http://www.mospi.nic.in
	Gaseous carbon emission and solid waste	The composition of carbon emissions data is derived from Climate Change Agenda for Delhi 2009-12, the emission data from industries are from GHG Platform India and Government of Delhi. Solid waste data are from Department of Environment, Government of National Capital Territory of Delhi (http://environment.delhigovt.nic.in).	Department of Environment, Government of Delhi[35]; Singh and Sharma[36] GHG Platform India (http://www.ghgplatform-india.org/industry-sector) Government of National Capital Territory of Delhi (http://delhi.gov.in) Solid waste (http://www.delhi.gov.in/wps/wcm/connect/environment/Environment/Home/Environmental+Issues/Waste+Management)
Hong Kong, China	Material imports/exports and stocks in products	Material imports and exports are compiled based on year 2006 and are decomposed into a finer sectoral level based on official records. The stock data are adjusted from a consultation with local researchers in the field of urban metabolism.	Trade and Industry Department, the Government of Hong Kong Special Administrative Region[37] Warren-Rhodes and Koenig[38] Langston et al[39]
	Energy supplied and consumed	Electricity generated inside and outside urban boundaries are distinguished based on data of thermal power plants of Hong Kong.	Census and Statistics Department of Hong Kong[40] https://www.censtatd.gov.hk/hkstat/sub/so90.jsp Ho and Siu[41] CCBF[42] World Bank[43]
	Gaseous carbon emission and solid waste	Carbon emission is calculated by this study considering emission from energy and industrial activities.	/
London, UK	Material imports/exports and stocks in products	Data of material imports/exports and sector contributions are from official reports including European Environment Agency and Government Office for Science of UK. Household stocks are based on 2002 urban metabolism inventory.	Institution of Wastes Management[44] EEA(European Environment Agency)[45] https://www.eea.europa.eu/publications/eea_report_2008_6 Government Office for Science, UK:https://assets.publishing.service.gov.uk/government/uploads/system/uploads/attachment_data/file/470766/gs-

			15-30-future-cities-urban-metabolism.pdf
	Energy supplied and consumed	Data are directly from sectoral energy flow inventory in statistics reported by City of London.	EEA (European Environment Agency)[46,47] Understanding the Data, City of London: https://www.london.ca/residents/Environment/Energy/Documents/Understanding_the_Data.pdf
	Gaseous carbon emission and solid waste	Carbon emission data are from statistics reported by City of London. Solid waste data are based on 2002 urban metabolic inventory.	EEA(European Environment Agency)[46,47]
Los Angeles, US	Material imports/exports and stocks in products	Material imports and exports are adjusted from urban inventory data in 2000, which is constrained by updated totals in 2008.	Ngo and Pataki[48] California Energy Commission[49] US Census Bureau[50]
	Energy supplied and consumed	Data are directly from official statistics of Los Angeles and US.	US Census Bureau[50] California Energy Commission[51] https://www.eia.gov/state/?sid=CA
	Gaseous carbon emission and solid waste	Data are directly from official statistics of Los Angeles and US. Emissions from electricity generated from inside and outside city boundary is distinguished vis grid data.	California Energy Commission[49] US Census Bureau[50] California Energy Commission[51]; Detailed electricity data: https://www.electricitylocal.com/states/california/los-angeles/
Moscow, Russia	Material imports/exports and stocks in products	Imports data of food are downscaled from national level according to population. Construction materials are downscaled based on sector's proportional GDP. Other data are from a survey of megacities accomplished by global researches (including our own work). Recycling rate is estimated based on national average.	Russia Federation[52] Paiho et al.[53] City of Moscow[54] Food consumption: FAO Food Balance Sheets (http://www.fao.org/statistics/en/ https://metabolismofcities.org/casestudy/3
	Energy supplied and consumed	Primary energy and electricity consumption and distribution data are from a survey of megacities accomplished by global researches (including our own work).	https://metabolismofcities.org/casestudy/3
	Gaseous carbon emission and solid waste	Calculated based on energy inventory of the city of Moscow. The emission from industrial process are not included due to a lack of such data.	City of Moscow[54] Brady McNall City of Moscow Sustainability Intern[55] Tkachenko and Tkachenko[56]

New York, US	Material imports/exports and stocks in products	Main data are from the survey of megacities accomplished by global researches (including our own work), and Department of Environmental Protection for New York City. Recycling of materials use inventory data in 2007.	US Census Bureau[50] PlaNYC[57] https://metabolismofcities.org/casestudy/22 http://www.dec.ny.gov/
	Energy supplied and consumed	Official statistics of New York City	NYSERDA[58,59]
	Gaseous carbon emission and solid waste	Emission and solid waste data are from the survey of megacities accomplished by global researches (including our own work).	US Census Bureau[60] City of New York[61]
Sao Paulo, Brazil	Material imports/exports and stocks in products	Consumption data are from published works and the survey of megacities accomplished by global researches (including our own work). Exports data are from Government of Sao Paulo.	Campillo et al.[15] Government of Sao Paulo: http://www.capital.sp.gov.br/portal/ https://metabolismofcities.org/casestudy/6
	Energy supplied and consumed	Published reports from UN and ICLEI	United Nations[62] ICLEI[63]
	Gaseous carbon emission and solid waste	Sectoral decompositions of carbon emissions are collected from C40 Cities Climate Leadership Group and ICLEI. Solid waste amount and composition data are from International Solid Waste Association (ISWA).	ICLEI[63] World Bank[64] C40 Cities Climate Leadership Group: https://www.c40.org/ ISWA: https://www.iswa.org
Singapore, Singapore	Material imports/exports and stocks in products	Construction and manufacturing sector Material flow data are from published literature, and food consumed by households are from FAO.	Chertow et al.[65] Schulz[66,67] Republic of Singapore[68] The City of Singapore[69] Food consumption: FAO Food Balance Sheets: http://www.fao.org/statistics/en/
	Energy supplied and consumed	Official energy statistics of the city of Singapore	Republic of Singapore[68] The City of Singapore[69]
	Gaseous carbon emission and solid waste	Own calculation based on official energy statistics of the city of Singapore	Chertow et al.[65] The City of Singapore[69]
Stockholm, Sweden	Material imports/exports and stocks in products	Import and export data are from European Environment Agency and the report from European Green Capital, and the changes in stock are complied based on published literature of urban metabolism.	Bringezu et al.[70] EEA[45] EEA[47] City of Stockholm[71,72] Stockholm Statistics-First European Green Capital: https://international.stockholm.se/city-

			development/european-green-capital-2010/
	Energy supplied and consumed	Official inventory reported by the City of Stockholm	City of Stockholm[71,72]
	Gaseous carbon emission and solid waste	Total carbon emissions and solid waste data are from the European Green City Index Report, while structure in 1995 is used for sector decomposition.	European Green City Index Report: https://www.siemens.com/entry/cc/features/greencityindex_international/all/en/pdf/stockholm.pdf
Sydney, Australia	Material imports/exports and stocks in products	Material imports are from Department of the Environment and Energy, the City of Sydney, and the sector contribution is estimated based on the industrial structure in 1990 (in which year such data is accessible).	Newman[73] SGS[74] Sydney Water [75] Department of the Environment and Energy: http://www.environment.gov.au/climate-change/government/carbon-neutral/publications/factsheet-city-of-sydney.
	Energy supplied and consumed	Energy use data at sector level are collected from Department of the Environment and Energy and Australian Energy Statistics.	City of Sydney[76] Australian Government Publishing Service[77] Lenzen et al.[78]
	Gaseous carbon emission and solid waste	Carbon emissions are calculated by this study from the energy use data and industrial activities.	/
Tokyo, Japan	Material imports/exports and stocks in products	Basic material flows data are extracted from Bureau of Environment of Tokyo. Household stocks and local supply are from the consultation with an urban metabolism research team University of Tokyo.	Bureau of Environment, Tokyo Metropolitan Government[79] Fujita and Hill[80] Bureau of Environment, Tokyo Metropolitan Government[81] Dhakal and Kaneko[82]
	Energy supplied and consumed	Official data reported by Bureau of Environment, Tokyo Metropolitan Government	Bureau of Environment, Tokyo Metropolitan Government: http://www.kankyo.metro.tokyo.jp/en/climate/index.html
	Gaseous carbon emission and solid waste	Carbon emissions are from official data reported by the of Tokyo. Solid waste data at sector level are then adjusted from big metropolitan area by its proportional GDP by sector.	Bureau of Environment, Tokyo Metropolitan Government
Toronto, Canada	Material imports/exports and stocks in products	Imports and exports data are compiled using the sectoral structure in 1999. The material flows of the core urban area are adjusted from metropolitan area with its proportional GDP by sector.	Sahely et al.[83] Forkes[84] Environment & Energy Division, City of Toronto[85]

	Energy supplied and consumed	Official data from Environment & Energy Division, City of Toronto	Environment & Energy Division, City of Toronto[85,86] Energy Efficiency Office, City of Toronto[87]
	Gaseous carbon emission and solid waste	Carbon emissions are calculated by this study based on energy use and industrial activities data.	Environment & Energy Division, City of Toronto [85,86] Energy Efficiency Office, City of Toronto[87]
Vienna, Austria	Material imports/exports and stocks in products	The imports and exports of goods in Vienna in 2005 are estimated from published literature of urban metabolism on Vienna, assuming the sector structure has remained the same. Part of the stock data are from European Environment Agency.	Obernosterer et al.[88] Hendriks[89] EEA[45,47]
	Energy supplied and consumed	Official data from the Energy Report of City of Vienna	EEA[45,47] Federal Environment Agency [90] City of Vienna[91]: https://www.wien.gv.at
	Gaseous carbon emission and solid waste	Carbon emissions are calculated by this study based on official data on energy use and industrial activities.	/

Data obtained from records of the city or local researchers

Data from publications/reports of the same boundary but are not fully-verified

Data extrapolated from the national- or regional-scale to the city-scale based on ratios

Supplementary Table 4 Sectoral value added used for calculating LQs of the 9 cities

	Sectoral value added (billion US$)						
City	Ag	Mi	Ma	En	Co	Tr	Se
Bangkok	7.1	1.9	25.8	5.3	3.6	11.1	33.8
Cape Town	1.0	0.7	8.3	2.4	3.1	6.4	33.0
Delhi	3.1	2.5	15.5	6.8	14.1	11.3	87.8
Moscow	19.2	9.6	57.9	24.3	12.2	28.2	169.6
Sao Paulo	11.3	17.1	67.5	29.5	24.4	26.0	212.6
Stockholm	0.4	0.1	5.4	1.0	1.7	2.0	23.5
Tokyo	8.2	2.6	72.4	15.3	28.1	72.4	311.1
Toronto	4.4	0.0	28.0	4.8	9.8	17.8	143.4
Vienna	1.1	1.2	12.6	3.2	4.8	4.1	58.5

Supplementary Table 5 Uncertainties of virtual carbon flow results considering carbon intensities and IO table rebalancing

City		ICF	HG	CF	EP
Bangkok	RSD(-)	-11%	-10%	-25%	-19%
	RSD(+)	11%	10%	25%	19%
Beijing	RSD(-)	-1%	-2%	-1%	-2%
	RSD(+)	1%	2%	1%	2%
Cape Town	RSD(-)	-28%	-29%	-24%	-23%
	RSD(+)	28%	29%	24%	23%
Delhi	RSD(-)	-12%	-16%	-23%	-17%
	RSD(+)	12%	16%	23%	17%
Hong Kong	RSD(-)	-5%	-2%	-3%	-6%
	RSD(+)	5%	2%	3%	6%
London	RSD(-)	-9%	-5%	-5%	-10%
	RSD(+)	9%	5%	5%	10%
Los Angeles	RSD(-)	-5%	-3%	-3%	-5%
	RSD(+)	5%	3%	3%	5%
Moscow	RSD(-)	-13%	-20%	-19%	-21%
	RSD(+)	13%	20%	19%	21%
New York	RSD(-)	-3%	-7%	-2%	-8%
	RSD(+)	3%	7%	2%	8%
Sao Paulo	RSD(-)	-3%	-30%	-17%	-25%
	RSD(+)	3%	30%	17%	25%
Singapore	RSD(-)	-12%	-22%	-27%	-26%
	RSD(+)	12%	22%	27%	26%
Stockholm	RSD(-)	-31%	-25%	-23%	-25%
	RSD(+)	31%	25%	23%	25%
Sydney	RSD(-)	-1%	-6%	-7%	-4%
	RSD(+)	1%	6%	7%	4%
Tokyo	RSD(-)	-14%	-9%	-1%	-13%
	RSD(+)	14%	9%	1%	13%
Toronto	RSD(-)	-10%	-19%	-9%	-24%
	RSD(+)	10%	19%	9%	24%
Vienna	RSD(-)	-14%	-20%	-8%	-25%
	RSD(+)	14%	20%	8%	25%

* These values fall into a 95% confidence interval.

Supplementary Table 6 Uncertainties of physical carbon flow results

City		IM	LS	RE	HS	GE	EX	SW	SC
Bangkok	RSD(-)	-8%	-6%	-8%	-9%	-6%	-12%	-11%	-19%
	RSD(+)	8%	6%	8%	9%	6%	12%	11%	19%
Beijing	RSD(-)	-18%	-6%	-10%	-11%	-4%	-19%	-16%	-5%
	RSD(+)	18%	6%	10%	11%	4%	19%	16%	5%
Cape Town	RSD(-)	-17%	-7%	-7%	-15%	-15%	-4%	-14%	-9%
	RSD(+)	17%	7%	7%	15%	15%	4%	14%	9%
Delhi	RSD(-)	-16%	-6%	-10%	-13%	-6%	-11%	-7%	-14%
	RSD(+)	16%	6%	10%	13%	6%	11%	7%	14%
Hong Kong	RSD(-)	-15%	-8%	-7%	-11%	-4%	-10%	-7%	-13%
	RSD(+)	15%	8%	7%	11%	4%	10%	7%	13%
London	RSD(-)	-6%	-5%	-8%	-7%	-7%	-7%	-8%	-6%
	RSD(+)	6%	5%	8%	7%	7%	7%	8%	6%
Los Angeles	RSD(-)	-15%	-6%	-9%	-10%	-7%	-10%	-17%	-26%
	RSD(+)	15%	6%	9%	10%	7%	10%	17%	26%
Moscow	RSD(-)	-20%	-7%	-13%	-19%	-12%	-12%	-18%	-11%
	RSD(+)	20%	7%	13%	19%	12%	12%	18%	11%
New York	RSD(-)	-16%	-5%	-11%	-13%	-6%	-8%	-7%	-8%
	RSD(+)	16%	5%	11%	13%	6%	8%	7%	8%
Sao Paulo	RSD(-)	-8%	-6%	-7%	-9%	-14%	-1%	-2%	-9%
	RSD(+)	8%	6%	7%	9%	14%	1%	2%	9%
Singapore	RSD(-)	-7%	-3%	-8%	-6%	-4%	-3%	-4%	-11%
	RSD(+)	7%	3%	8%	6%	4%	3%	4%	11%
Stockholm	RSD(-)	-8%	-6%	-3%	-12%	-9%	-1%	-3%	-14%
	RSD(+)	8%	6%	3%	12%	9%	1%	3%	14%
Sydney	RSD(-)	-13%	-7%	-7%	-1%	-10%	-1%	-6%	-5%
	RSD(+)	13%	7%	7%	1%	10%	1%	6%	5%
Tokyo	RSD(-)	-11%	-9%	-9%	-6%	-4%	-2%	-6%	-6%
	RSD(+)	11%	9%	9%	6%	4%	2%	6%	6%
Toronto	RSD(-)	-11%	-7%	-9%	-12%	-7%	-6%	-8%	-11%
	RSD(+)	11%	7%	9%	12%	7%	6%	8%	11%
Vienna	RSD(-)	-10%	-5%	-6%	-10%	-8%	-2%	-6%	-8%
	RSD(+)	10%	5%	6%	10%	8%	2%	6%	8%

* These values fall into a 95% confidence interval.

Supplementary Table 7 Control of uncertainties in the integrated model

Source of uncertainty	Influenced aspect	Way of control
Variation between calorific value of local fuel types and that derived from IPCC	Physical carbon flow	We use calorific values related to the local area where possible. For example, for Beijing, we use Chinese specific calorific value of different fuel types (often lower than IPCC defaults)
Material flow data from various sources	Physical carbon flow and virtual carbon flow	Where sector-level material data are not available, we use the energy and material data of published literature (often in total) to restrain the sectoral decomposed metabolism data. But uncertainty do exist since there is no actual values to compare with. Different types of data sources for cities are described in Supplementary Table 4.
Recycling rates of materials	Physical carbon flow	We recognize some of informal recycling activities of materials could be missed in the model due to a lack of data in many cities (especially cities in developing countries), but this will not have major effect on the total physical inflow.
Downscaling IO table for urban economies	Virtual carbon flow	We combine a standardized LQ and CIQ method from applied regional analyses to derive urban input-output tables. Value added (urban GDP) and total income are used as main constrains to better reflect local economies.
RAS technique used in balancing urban IO tables	Virtual carbon flow	We apply a refined RAS approach based on the work of Lenzen et al.[92] to cope with conflicts of information.
The assumptions of homogeneity and production technology inherent in input-output models	Virtual carbon flow	This is an inherent uncertainty in all input-output models. IOA assumes the homogeneity of activities within a sector, which could lead to uncertainties in delineating activities of the economy. Also, the production technology of a sector is often assumed to be constant in the technical structure.
Justification of urban vegetation and sequestration rates	Carbon sinks	We estimate the carbon sequestration rates of urban trees based on their forest coverage and city-specific reference values of carbon sequestration rate from literature. The uncertainties in natural sequestration are given.

Supplementary Notes

Supplementary Note 1

Scientists have developed a range of accounting frameworks to capture material flows through the human economy at multiple scales[93-104]. Most of these accounting frameworks and approaches can be adopted to city-scale analysis, albeit some of them are originally designed for economies at larger spatial scales such as regions or countries.

Here we use a metabolism-based framework to account for physical carbon inputs to, stored in and leaving urban areas. It should also be noted that for urban carbon accounting we are using citywide material flow data rather than down-scaled national material flow data in most cases. All carbon flows of the urban economies are assessed via the aggregate economic sectors: Agriculture (Ag), Mining (Mi), Manufacturing (Ma), Supply of electricity, gas and hot water (En), Construction (Co), Transportation (Tr), and Services (Se). Note that the service sector (Se) includes a range of activities such as retail trade, hotels and catering service, leasing and business services, and research development. The accounting approaches of urban physical carbon flows are explained in the following:

I. Accounting of physical carbon. In terms of material flows of cities, the most reliable data sources are provided by urban metabolism studies based on material or substance flow analysis[105,106] A number of field surveys and accounting of materials and energy flows of specific cities have been conducted under this framework[107-109]. We collect the most recent and reliable data for the selected cities from official statistics and reports and published literature (the sources of material and energy flows for each city are listed in Supplementary Table 4). They are compiled using a consistent integrated framework (Figure 1 in the main text). The recycling of materials includes products such as wood, steel, paper, etc. after their first use (excluding methane emitted from waste due to a lack of data for most cities), which is derived from a survey of recycled solid waste for each city. Household retention of physical carbon (HS) only include carbon stored in households usually for more than one year (such as furniture, book and other durable products). Solid waste accounts for both carbon in industrial waste and that in less durable household products such as foods. Waste data are acquired from multiple sources such as urban MFA studies, city-level statistical yearbooks, or Eurostat environmental database[110-120]. though the electricity use data was collected in this section, it was not considered in the physical carbon flows but was included in the virtual carbon flows. Among all the material imports, the import of food for residential consumption is derived from city-scale data from the literature, as have been done in many metabolism studies (e.g.[121-124]).

For food processing in industrial sector and food consumption by tourists (service sector), Beijing, Hong Kong, London, Vienna, Stockholm, Singapore, Sydney and Paris have official data. For other cities that do not have this data, the food possessing in the industrial sector and food consumption by tourists (commercial and service sector) are scaled from national data (FAOSTAT database[125] and national statistical sources) using the share of the urban sector's economic output relative to the respective sector's national output[126,127].

II. Similar to the practices in literatures[128-130], we convert mass-based flows to carbon flows by multiplying them with the carbon content factor (CCF). In this case, sector-specific CCF (CCF^i) is calculated from the aggregation of the product-specific CCF (CCF_p^i):

$$CCF^i = \sum_{p=1} CCF_p^i M_p^i \Big/ \sum_{p=1} M_p^i \quad \text{(S1)}$$

in which *p* is a certain type of product within Sector *i;* M_p^i is the corresponding weight of that product. The products and raw materials varied in terms of the different types of products, therefore extensive literature research is conducted to obtain product-based CCFs of fuel and biomass[129,131-135], agricultural and food products[136-138], and industrial and construction materials[139-141].

III. Natural sequestration refers to the sequestration of carbon dioxide from the atmosphere. Urban vegetation has been reported as a major source for carbon sequestration in cities[142,143], although others doubt its significance and effectiveness as global carbon sinks[144]. We estimate the capacity for carbon sequestration by trees based on their forest coverage and reference values of carbon sequestration rate for each city[145-149]. Note that urban soils are not considered for natural carbon sequestration in this study. In comparison to vegetation, the carbon sequestration effect by urban soils is more complex and uncertain[150,151]. Other studies have shown its insignificance compared to sequestration by vegetation[152,153].

IV. Due to the lack of city-level IO tables, there have been studies of coupling traditional methods of urban metabolism (such as MFA) with life cycle analysis (LCA) to advance the understanding of urban carbon flows (e.g.[154,155]). A number of other studies have proposed a cross-boundary quantification approach for urban metabolism by integrating MFA and LCA into environmental impact assessment of cities[156,121]. We adopted this MFA-LCA integrated method to calculate the carbon emission embodied in the imports to the global cities, with adjustments on the sector categorization according to the data framework of material flows. The carbon emission coefficients of material and energy inputs are mainly derived from EcoInvent 2.01 database[157] and are supplemented with processes from the built-in professional database in Simapro 7 when the EcoInvent data do not match with the cities. In addition, city-specific situations of

technology and setting are taken into consideration. The industrial processes of producing agro-products and electronic products, etc. are highly associated with the technology related to each city. For example, different emission factors for coal-power, wind-power and nuclear-power electricity used by cities were used. In order to examine how the embodied emissions are driven by final uses of the urban economy (household and government consumption, capital formation, exports, which is consumption-based emission), we applied input-output analysis (IOA)[158,159] in the allocation process.

Supplementary Note 2

Beijing, Hong Kong, Singapore, Sydney, London, New York and Los Angeles have city-level IO tables that can be readily used (IO tables of New York and Los Angeles are derived from IMPLAN, a regional table complication technique for US cities). We compile urban IO tables for the rest cities in this study. The absence of urban input-output (IO) tables has been a main suppression in modelling virtual carbon flows (or other kinds of embodied flows) at city level. Nonetheless, the need of measuring urban carbon balances from physical and virtual perspectives is huge given the high linkage of urban carbon profile with its external markets (or hinterlands). Some important progresses have been made to disaggregate national IO tables to smaller scales such as regions and cities[160]. Some established approaches include IMPLAN (impact analysis for planning), RIMS I, RIMS II (Regional Impact Modeling System). These approaches often use location quotients (LQ) and cross-industry quotients (CIQ) to derive estimates of regional input coefficients, widely accepted and applied in regional economic and environmental analyses.

Here we combine a standardized LQ and CIQ method from applied regional analyses to derive urban input-output tables for cities lacking such data. There are two reasons for applying this technique: (1) It allows for intensive cell-by-cell adjustments for non-diagonal elements and uniform adjustments for diagonal elements in the monetary flow matrix; (2) The relative weight of both selling sector *i* and buying sector *j* in the region and the nation is considered, which is important when the scale of the targeted economy is relatively small (such as a city).

The location quotients (LQ) and cross-industry quotients (CIQ) are defined as[160]:

$$LQ_i^u = \frac{\mathrm{x}_i^u (\mathrm{x}_i^n)^{-1}}{\mathrm{x}^u (\mathrm{x}^n)^{-1}} \tag{S2}$$

$$CIQ_{ij}^u = \frac{\mathrm{x}_i^u (\mathrm{x}_i^n)^{-1}}{\mathrm{x}_j^u (\mathrm{x}_j^n)^{-1}} \tag{S3}$$

where x_i^n and x_i^u are the total output of selling sector *i* in national economy and targeted

urban economy, respectively; x_j^n and x_j^u are the total output of buying sector *j* in national economy and targeted urban economy, respectively. x^u and x^n is the total output of entire national economy and targeted urban economy, respectively. The input coefficients of the urban economy (a_{ij}^u) are then estimated from LQ, CIQ and input coefficients of the national economy (a_{ij}^n):

$$a_{ij}^u = \begin{Bmatrix} (CIQ_{ij}^u)a_{ij}^n & \text{if } CIQ_{ij}^u < 1 \\ a_{ij}^n & \text{if } CIQ_{ij}^u \geq 1 \end{Bmatrix} \text{ for } i \neq j \text{ (non-diagnal elements)}$$
$$a_{ij}^u = \begin{Bmatrix} (LQ_i^u)a_{ij}^n & \text{if } LQ_i^u < 1 \\ a_{ij}^n & \text{if } LQ_i^u \geq 1 \end{Bmatrix} \text{ for } i = j \text{ (diagnal elements)} \quad \text{(S4)}$$

RAS technique is used to balance the urban IO tables. This technique has been widely used as an automatic technique in updating IO tables. The process in RAS can be seen as an iterative scaling of a non-negative matrix until its column sums and row sums equal given vectors. The detailed iterative process has been described in references[92,160,]. We use local total outputs and value added in the corresponding year as the main constraints in balancing the tables.

To minimize the possible distortion of urban carbon modelling results, (1) IO tables are amended with high-quality local inventory data of sector level-total outputs; (2) Local value added and final consumption expenditure of from urban database are used as main constrains for table balance and calibration; (3) life-cycle carbon emissions from each urban sector are used to calculate carbon intensities rather than national averages, which is based on real inventory data of key materials and products consumed by the cities.

It should be noted that despite all the efforts we have made to minimize uncertainty from urban input-output table, not all sections of the input-output tables are fully verifiable. In essence, flows like value added, final consumption, exports are verified based on cities' statistical record while intermediate flows can't be fully verified given the lack of survey data. Miller and Blair[160] pointed out that table disaggregation approaches (such as LQ and CLQ) are frequently used in applied regional analysis, but we are quite clear they should only be used to provide broad insights of problems. We do not intend to establish accurate and fully-verified IO tables for global cities. Instead, we aim to relate cities' final demands to their different carbon inflows, and to demonstrate the importance of urban virtual carbon flows.

Supplementary Note 3

The uncertainties of the model and findings are estimated when possible (as shown in

Supplementary Figure 3). For physical carbon, the uncertainties in calculating carbon content in materials caused by the selection of carbon content factors (CCFs) are determined for physical carbon inflows to a city. The lower and upper ranges of the results of physical carbon flows are determined based on a range of estimated values of CCFs. For virtual carbon, there are two situations in determining uncertainties of the model used that should be treated differently. Regarding cities that already have available urban level input-output tables (i.e. Beijing, Hong Kong, Singapore, London, Sydney, New York and Los Angeles), we assume the uncertainties mainly come from the calculation of direct carbon emission intensities for sectors. For the other 9 cities that do not have official input-output tables, the uncertainties come from the accumulation of two modelling steps, i.e. estimation of carbon intensities calculation and rebalancing of urban IO tables. These uncertainties are quantified for the 9 cities, while the impact of downscaling national IO tables is only qualitatively described. Finally, we have provided a description of raw data sources in Supplementary Table 4 to report the possible data uncertainty.

In terms of Monte Carlo analyses of input–output systems, standard deviations (SDs) were generally used in literature (e.g.[161,162]). Lenzen and colleagues[163] calculated the SDs form input-output modelling process to test their findings of UK's carbon footprint. Here we use a similar approach to determine the uncertainties of carbon flows modelling for cities. The uncertainties of physical carbon flows (PCF) and virtual carbon flows (VCF) considered in this study can be formulated as:

$$PCF^{*}=M \times CCF^{*} \tag{S5}$$

$$\sigma_p = \sqrt{\frac{\sum_{i=1}^{N}(PCF^{*}\text{-}PCF)^2}{N-1}} \tag{S6}$$

$$RSD_p = \frac{\pm 2\sigma_p}{PCF} \tag{S7}$$

$$VCF^{*}=k^{*}(\text{I-A}^{*})^{-1}y^{*} \tag{S8}$$

$$\sigma_v = \sqrt{\frac{\sum_{i=1}^{N}(VCF^{*}\text{-}VCF)^2}{N-1}} \tag{S9}$$

$$RSD_v = \frac{\pm 2\sigma_v}{VCF} \tag{S10}$$

where *PCF* and *VCF* represent the values of various physical and virtual carbon flows, respectively; *k* is the carbon intensity; *y* is urban final demand; the asterisk (*) represent the returns to each possible result of modelling. This can be used to determine upper and lower bounds of the modelling results since all the PCF results are expected to be within the range of [PCF–2σ_p, PCF+2σ_p] with a 95% confidence. Similarly, all the VCF results are expected to be within the range of [VCF–

$2\sigma_p$, VCF+$2\sigma_p$] with a 95% confidence, in which the uncertainty of carbon intensities and the impact of IO table rebalancing on the technical coefficient matrix (A) and final demand (y) are considered. Here PCF refers to results related to inflows such as imports from other regions (IM), local supply by urban ecosystems (LS) and recycling of materials (RE), as well as all outflows including household storage (HS), solid waste (SW), and export to external markets (EX) and changes in carbon stock (SC). Gaseous emission (CO_2) within urban territory has been verified for all cities, and therefore is considered accurate. VCF refers to results of import carbon emission (ICF), and emissions embodied in household consumption (HG), capital formation (CF) and export (EP). RSD_p and RSD_v (relative standard deviation, usually in ±%) are the ratios of standard deviations to total value of PCF and VCF, respectively.

Supplementary References

1. Food and Agricultural Organization of the United Nations 2012 FAOStat (available at: http://faostat3.fao.org) (2012)
2. Roy P, Nei D, Orikasa T, et al. A review of life cycle assessment (LCA) on some food products. Journal of Food Engineering, 2009, 90(1): 1-10.
3. Garnett T. Livestock-related greenhouse gas emissions: impacts and options for policy makers. Environmental Science & Policy, 2009, 12(4): 491-503.
4. Perugini F, Mastellone M L, Arena U. A life cycle assessment of mechanical and feedstock recycling options for management of plastic packaging wastes. Environmental Progress, 2005, 24(2): 137-154.
5. Fay R, Treloar G, Iyer-Raniga U. Life-cycle energy analysis of buildings: a case study. Building Research & Information, 2000, 28(1): 31-41.
6. Rafenberg C, Eric M. Life cycle analysis of the newspaper Le Monde. The International Journal of Life Cycle Assessment, 1998, 3(3): 131-144.
7. Aoe T. Eco-efficiency and ecodesign in electrical and electronic products. Journal of Cleaner Production, 2007, 15(15): 1406-1414.
8. Teehan P, Kandlikar M. Comparing embodied greenhouse gas emissions of modern computing and electronics products. Environmental Science & Technology, 2013, 47(9): 3997-4003.
9. Hertwich E G, Roux C. Greenhouse gas emissions from the consumption of electric and electronic equipment by Norwegian households. Environmental Science & Technology, 2011, 45(19): 8190-8196.
10. Mehta, VK, et al., 2014. Urban metabolism in Bangalore and Bangkok: findings and future directions 2014 SEI science forum, Stockholm Sweden.
11. National Statistical Office of Thailand. Environment and Energy Statistics (2008). http://web.nso.go.th/.
12. Murakami, A., Medrial, Zain A., Takeuchi, K., Tsunekawa, A., Yokota, S. Trends in urbanization and patterns of land use in the Asian mega cities Jakarta, Bangkok, and Metro Manila. *Landscape and Urban Planning* 70, 251-259 (2005).

13. Phdungsilp A. Energy Analysis for Sustainable Mega-Cities. P106-141 (2006).
14. Phdungsilp A. Integrated energy and carbon modeling with a decision support system: Policy scenarios for low-carbon city development in Bangkok. *Energy Policy*, 38(9), 4808-4817 (2010).
15. Campillo, G., et al. Mainstreaming Urban Metabolism: Advances and challenges in city participation. Sixth Urban Research and Knowledge Symposium 2012 (2012).
16. Beijing Municipal Bureau of Statistics. Beijing Statistic Yearbook 2008. Beijing: China Statistics Press (2009) (in Chinese).
17. Editorial Board of China Commerce Yearbook. China Commerce Yearbook (2008). China Commerce Press, Beijing (2009) (in Chinese).
18. Editorial Board of China Iron and Steel Industry Yearbook. China Iron and Steel Industry Yearbook 2008. Metallurgy Industry Publisher, Beijing (2009) (in Chinese).
19. Editorial Board of China Mining Industry Yearbook. China Mining Industry Yearbook 2008. Earthquake Press, Beijing (2009) (in Chinese).
20. State Environmental Protection Administration of China. Annual Statistic Report on Environment in China 2008. China Environmental Science Press, Beijing (2009) (in Chinese).
21. Beijing Municipal Bureau of Statistics, Beijing Statistic Yearbook 2008. Beijing: China Statistics Press (in Chinese) (2009)
22. Beijing Statistics Bureau. Beijing: China Statistics Press (2008).
23. Lu W, et al., Urban energy consumption and related carbon emission estimation: a study at the sector scale. *Front. Earth Sci.* 7, 480–486 (2013)
24. Feng Y Y, Chen S Q, Zhang L X. System dynamics modeling for urban energy consumption and CO2 emissions: a case study of Beijing, China. *Ecol Modell*, 252, 44–52 (2012).
25. Department of Economic and Human Development, City of Cape Town. The Economic Imperatives of Environmental Sustainability (2007).
26. Campillo, G., et al. Mainstreaming Urban Metabolism: Advances and challenges in city participation. Sixth Urban Research and Knowledge Symposium 2012 (2012).
27. Swilling M. Sustainability and infrastructure planning in South Africa: a Cape Town case study. Environment and Urbanization, 18, 23-50 (2006).
28. Ward S, Walsh V. Cape Town energy case study. Environmental Resource Management Department //World Energy Congress. (2010).
29. City of Cape Town. State of the Environment Report 2007/08 (2008)
30. City of Cape Town. 5 Year Plan for Cape Town; Integrated Development Plan (IDP) 2007/8 – 2011/12. (2008)
31. Delhi Statistical Handbook 2010. http://www.delhi.gov.in/
32. Ruet J, Zerah M, Saravanan V S. The water and sanitation scenario in Indian metropolitan cities: resources and management in Delhi, Calcutta, Chennai, Mumbai. (2009).
33. Streicher-Porte M, Widmer R, Jain A, et al. Key drivers of the e-waste recycling system: Assessing and modelling e-waste processing in the informal sector in Delhi. *Environmental Impact Assessment Review*, 25, 472-491 (2005).
34. Central Statistics Office, Ministry of Statistics and Programme Implementation Government of India, New Delhi. Energy Statistics. http://www.mospi.nic.in.
35. Department of Environment, Government of Delhi. Executive Summary of Inventorization of

Green House Gases – Sources and Sinks in Delhi (2008).

36. Singh R, Sharma C. Assessment of emissions from transport sector in Delhi. *Journal of Scientific & Industrial Research* 71, 155-160 (2012).

37. Trade and Industry Department, the Government of Hong Kong Special Administrative Region (2007). http://www.tid.gov.hk/english.

38. Warren-Rhodes K, Koenig A. Escalating trends in the urban metabolism of Hong Kong: 1971-1997. *AMBIO*, 30, 429-438 (2001).

39. Langston C, Wong F K W, Hui E, et al. Strategic assessment of building adaptive reuse opportunities in Hong Kong. *Building and Environment*, 43(10), 1709-1718 (2008).

40. Census and Statistics Department of Hong Kong. Hong Kong Energy Statistics 2006 Annual Report (2007). http://www.statistics.gov.hk/pub/B11000022006AN06B0100.pdf.

41. Ho CY, Siu KW. A dynamic equilibrium of electricity consumption and GDP in Hong Kong: an empirical investigation. Energy Policy, 35(4), 2507-2513 (2007).

42. CCBF (Climate Change Business Forum) (2008). http://www.climatechangebusinessforum.com/en-us/hong_kong_context_emissions

43. World Bank. Trading Economics dataset. (2008) http://www.tradingeconomics.com/hong-kong/co2-emissions-metric-tons-per-capita-wb-data.html

44. Institution of Wastes Management. Introducing city limits: a resource flow and ecological footprint analysis of Greater London. Best Foot Forward (2002).

45. EEA. Europe's environment — The fourth assessment. State of the environment report No 1/2007, (2007).

46. EEA. Ensuring quality of life in Europe's cities and towns. EEA Report No 5/2009 (2009).

47. EEA. Energy and environment report 2008. EEA Report No 6/2008 (2009).

48. Ngo N S, Pataki D E. The energy and mass balance of Los Angeles County. *Urban Ecosystems*, 11(2): 121-139 (2008).

49. California Energy Commission. City of Los Angeles. (2009) http://www.laalmanac.com/LA/index.htm.

50. US Census Bureau. Economic Census. (2009). http://www.census.gov/econ/census/.

51. California Energy Commission. Los Angeles Energy Consumption by End Use. (2009). http://energyalmanac.ca.gov/

52. Russia Federation. Federal State Statistics Service. http://www.gks.ru/. (2008)

53. Paiho S, Hoang H, Hedman Å, et al. Energy and emission analyses of renovation scenarios of a Moscow residential district. *Energy and Buildings*, 76: 402-413. (2014)

54. City of Moscow. Greenhouse Gas & Energy Efficiency Report 2010. (2010)

55. Brady McNall City of Moscow Sustainability Intern. City of Moscow Green House Gas Inventory Update. (2012)

56. Tkachenko S., Tkachenko L. Urban planning and climate change adaptation in Moscow city. Institute of Moscow City Master Plan (2010)

57. PlaNYC. A Greener, Greater New York, Mayor’s Office of Long Term Planning and Sustainability (OLTPS). (2007) http://www.nyc.gov/html/oltps/html/home/home.shtml

58. NYSERDA. New York State Energy Plan 2010. Volume 2. http://energyplan.ny.gov/ (2010).

59. NYSERDA. Patterns and Trends: New York State Energy Profiles (1997–2011). June 2013.
60. US Census Bureau. Economic Census. (2009) http://www.census.gov/econ/census/.
61. City of New York, Inventory of New York City Greenhouse Gas Emissions, September 2010, by Jonathan Dickinson and Rishi Desai. Mayor’s Office of Long-Term Planning and Sustainability, New York (2010).
62. United Nations. International cooperation of cities: the C-40 initiative. (2009)
63. ICLEI, 2008. São Paulo, Brazil: Turning pollution into profit: the Bandeirantes Landfill Gas to Energy Project.
64. World Bank. Sao Paulo case study overview: climate change, disaster risk and the urban poor: cities building resilience for a changing world. www.worldbank.org. (2010)
65. Chertow M, Choi E, Lee K. The material consumption of Singapore’s economy: An industrial ecology approach. Environment and climate change in Asia: Ecological footprints and green prospects. Pearson: Prentice Hall. (2011).
66. Schulz NB. The physical material inputs into Singapore's development. *Journal of Industrial Ecology*, **11**(2), 117-131(2007).
67. Schulz NB. Contributions of material and energy flow accounting to urban ecosystems analysis: Case study Singapore. UNU-IAS Working Paper, 2005, 136.
68. Republic of Singapore. National Climate Change Strategy 2012. (2012)
69. The City of Singapore. Singapore Energy Statistics 2007. (2008)
70. Bringezu S et al. (Eds). Analysis for Action: Support for Policy towards Sustainability by Material Flow Accounting. Proceedings of the ConAccount Conference 1997. (1998)
71. City of Stockholm. Energy Future of the Stockholm Region 2010-2050 Final Report. (2009)
72. City of Stockholm. City of Stockholm’s Climate Initiatives. (2010)
73. Newman P W G. Sustainability and cities: extending the metabolism model. *Landscape and Urban Planning*, **44**(4), 219-226(1999).
74. SGS. Australian Cities Accounts 2010-11 Estimates. (2010)
75. Department of the Environment and Energy, the City of Sydney. http://www.environment.gov.au/climate-change/government/carbon-neutral/publications/factsheet-city-of-sydney.
76. City of Sydney. State of the Environment Report 2011/12. (2012)
77. Australian Government Publishing Service. Energy and Environment Statiatics. (2008) http://australia.gov.au/topics/environment-and-natural-resources.
78. Lenzen M, Dey C, Foran B. Energy requirements of Sydney households. *Ecological Economics*, 49(3), 375-399 (2004).
79. Bureau of Environment, Tokyo Metropolitan Government. Comparison of Cities in the World (2005).
80. Fujita K, Hill R C. The zero waste city: Tokyo's quest for a sustainable environment. *Journal of Comparative Policy Analysis*, 9(4), 405-425(2007).
81. Bureau of Environment, Tokyo Metropolitan Government. Energy Consumption and Waste statisitcs (2009).
82. Dhakal, S., Kaneko, S. Urban Energy Use in Asian Mega-Cities: Is Tokyo a Desirable Model?

Proceedings of Workshop of IGES/APN Mega-City Project (2002).

83. Sahely H R, Dudding S, Kennedy C A. Erratum: Estimating the urban metabolism of Canadian cities: Greater Toronto Area case study. Canadian *Journal of Civil Engineering*, **30**(4), 794-794 (2003).

84. Forkes J. Nitrogen balance for the urban food metabolism of Toronto, Canada. *Resources, Conservation and Recycling*, 52(1), 74-94 (2007).

85. Environment & Energy Division, City of Toronto. Power to Live Green: Climate & Energy Goals (2010).

86. Environment & Energy Division, City of Toronto. Summary of Toronto's 2011 Greenhouse Gas and Air Quality Pollutant Emission Inventory. Staff Report (2011).

87. Energy Efficiency Office, City of Toronto. Energy Efficiency & Beyond: Toronto's Sustainable Energy Plan. Staff Background Report (2007).

88. Obernosterer, R.; Brunner, P.H.; Daxbeck, H.; et al. Material Accounting as a Tool for Decision Making in Environmental Policy−MAcTEmPo case study report−urban metabolism, the city of Vienna (Vienna, Institute for Water Quality and Waste Management, Technical University of Vienna) (1998).

89. Hendriks, C.; Obernosterer, R.; Muller, D.; Kytzia, S.; Baccini, P.; Brunner, P.H. Material flow analysis: a tool to support environmental policy decision making. Case studies on the city of Vienna and the Swiss lowlands. *Loc. Environ* 5, 311−328(2000).

90. Federal Environment Agency: Ninth Environment Report. Environmental situationin Austria. Reports, Vol REP-0286th Federal Environmental Agency, Vienna (2010).

91. City of Vienna. Energy Report of the City of Vienna. https://www.wien.gv.at

92. Lenzen M, Gallego B, Wood R. Matrix balancing under conflicting information. *Economic Systems Research*, 2009, 21(1): 23-44.

93. Kennedy C, Hoornweg D. Mainstreaming urban metabolism. Journal of Industrial Ecology, 16(6), 780-782 (2012).

94. Haberl H. The energetic metabolism of societies part I: Accounting concepts. *Journal of Industrial Ecology*, **5**, 11-33(2001).

95. Rosado L, Niza S, Ferrão P. A material flow accounting case study of the Lisbon metropolitan area using the urban metabolism analyst model. Journal of Industrial Ecology, 18(1), 84-101 (2014).

96. Decker E H, Elliott S, Smith F A, et al. Energy and material flow through the urban ecosystem. *Annual Review of Energy and The Environment*, 25, 685-740 (2000).

97. Brunner P H, Rechberger H. Practical handbook of material flow analysis. The International Journal of Life Cycle Assessment, 9(5), 337-338 (2004).

98. Piña W H A, Martínez C I P. Urban material flow analysis: An approach for Bogotá, Colombia. Ecological indicators, 42, 32-42 (2014).

99. Huang S L, Hsu W L. Materials flow analysis and emergy evaluation of Taipei's urban construction. Landscape and Urban Planning, 63(2), 61-74 (2003).

100. Liu, Z., K. Feng, K. Hubacek, et al., Four system boundaries for carbon accounts. Ecological Modelling 318, 118-125 (2015).

101. Ramaswami A, Chavez A, Chertow M. Carbon footprinting of cities and implications for analysis of urban material and energy flows. *Journal of Industrial Ecology*, 16(6), 783-785

(2012).

102. Mulalic, I. Material Flows and Physical Input-Output Tables-PIOT for Denmark 2002 based on MFA (Statistics Denmark, 2007).

103. Giljum, S., & Hubacek, K. Alternative approaches of physical input–output analysis to estimate primary material inputs of production and consumption activities. *Economic Systems Research*, **16**, 301-310(2004).

104. Wiedmann, T. O. *et al*. The material footprint of nations. *Proceedings of the National Academy of Sciences*, 201220362 (2013).

105. Wolman, A. The metabolism of cities. *Sci. Am.*, **213**, 179−190(1965).

106. Kennedy, C., Cuddihy, J. & Engel−Yan, J. The changing metabolism of cities. *Journal of Industrial Ecology*, **11**, 43−59 (2007).

107. Niza, S., Rosado, L., & Ferrao, P. Urban metabolism-methodological advances in urban material flow accounting based on the Lisbon case study. *Journal of Industrial Ecology* 13 (3), 384-405 (2009).

108. Warren-Rhodes, K. & Koenig, A. Escalating trends in the urban metabolism of Hong Kong: 1971−1997. *R. Swed. Acad. Sci.* **30**, 429−438 (2001).

109. Sahely, H.R., Dudding, S. & Kennedy, C.A. Estimating the urban metabolism of Canadian cities: GTA case study. *Canadian Journal for Civil Engineering,* **30**, 468-483(2003).

110. Murakami, A., Medrial, Zain A., Takeuchi, K., Tsunekawa, A., Yokota, S. Trends in urbanization and patterns of land use in the Asian mega cities Jakarta, Bangkok, and Metro Manila. *Landscape and Urban Planning* **70**, 251-259 (2005).

111. Phdungsilp, A. *Energy Analysis for Sustainable* Mega-*Cities* (Doctoral dissertation, KTH, Stockholm, Sweden, 2006).

112. Beijing Municipal Bureau of Statistics. *Beijing Statistical Yearbook 2006* (China Statistical Publishing House, Beijing, 2007).

113. Ward, S., Walsh, V. *City of Cape Town: Energy for Large Cities Report*. Energy and Climate Change Branch, World Energy Congress, 2010).

114. Chambers N, et al. *A Resource Flow and Ecological Footprint Analysis of Greater London* (Best Foot Forward Ltd, Oxford, 2002).

115. Ngo, N.S., Pataki, D.E. The energy and mass balance of Los Angeles County. *Urban Ecosyst* **11**, 121–139(2008).

116. Barles, S. Urban metabolism of Paris and its region. *Journal of Industrial Ecology* **13**, 898-913(2009).

117. Singapore Energy Market Authority (EMA). *Singapore Energy Statistics 2012*. (Singapore, 2012).

118. OECD. OECD Territorial Reviews: Toronto, Canada (2010).

119. Sydney Statistical Division. *Australian Bureau of Statistics 2005* (2005).

120. Eurostat. *European Commission.* http://epp.eurostat.ec.europa.eu/portal/page/portal/region_cities/city_urban/spatial_units (2012).

121. Goldstein B, Birkved M, Quitzau M B, et al. Quantification of urban metabolism through coupling with the life cycle assessment framework: concept development and case study. *Environmental Research Letters*, **8**(3), 035024 (2013).

122. Luo T W, Ouyang Z Y, Wang X K, et al. Dynamics of urban food-carbon consumption in Beijing households. Acta Ecologica Sinica, 2005, 25(12): 3252-3258.

123. Barles S. Feeding the city: food consumption and flow of nitrogen, Paris, 1801–1914. Science of the Total Environment, 2007, 375(1): 48-58.

124. Ma H, Huang J, Fuller F, et al. Getting rich and eating out: consumption of food away from home in urban China. Canadian Journal of Agricultural Economics/Revue canadienne d'agroeconomie, 2006, 54(1): 101-119.

125. FAO 2014 FAOSTAT Online Database (available at http://faostat. fao.org/, accessed September 2014).

126. Dawson, P.J., 1997. The demand for calories in developing countries. *Oxford Develop. Stud.* 25 (3), 361–369.

127. World Bank, 1986. Poverty and Hunger – Issues and Options for Food Security in Developing Countries. The World Bank, Washington DC. 69 pp.

128. Chen, S.Q. & Chen, B. Network environ perspective for urban metabolism and carbon emissions: A case study of Vienna, Austria. *Environmental Science & Technology*, **46**, 4498−4506 (2012).

129. Zhao R, Huang X, Zhong T, et al. Carbon flow of urban system and its policy implications: the case of Nanjing. *Renewable & Sustainable Energy Reviews*, 33, 589-601 (2014).

130. Hao, Y., Su, M., Zhang, L., Cai, Y. & Yang, Z. Integrated accounting of urban carbon cycle in Guangyuan, a mountainous city of China: the impacts of earthquake and reconstruction. J. Clean. Prod. 103, 231-240 (2015).

131. IPCC, Revised 1996 *Guidelines for National Greenhouse Gas Inventories: Workbook* (1997). http://www.ipcc-nggip.iges.or.jp/public/gl/invs1.html.

132. Australia Government Department of Environment (AGDE). *National Greenhouse and Energy Reporting (Measurement) Technical Guidelines 2012* (2012). http://www.climatechange.gov.au/sites/climatechange/files/documents/04_2013/nger-technical-guidelines-2012.pdf.

133. DAFF. *Australian State of the Forests Report: Five yearly report 2008*. Australian Bureau of Agricultural and Resource Economics and Sciences. Department of Agriculture, Forestry and Fisheries (2008).

134. Lamlom, S.H. & Savidge, R.A. A reassessment of carbon content in wood: variation within and between 41 North American species. *Biomass and Bioenergy* **25**, 381-388(2003).

135. Stockmann, K.D. *et al*. Estimates of carbon stored in harvested wood products from the United States forest service northern region, 1906-2010. *Carbon Balance and Management*, **7**, 1 (2012).

136. University of Michigan Health System. Diabetes: Carbohydrate Food List (2011).

137. FAO. The EX-Ante Carbon-balance Tool (EX-ACT) (2009). http://www.fao.org/tc/exact/carbon-balance-tool-ex-act/en/.

138. Moriarty D.J.W & Barclay, M.C. Carbon and Nitrogen Content of Food and the Assimilation Efficiencies of Penaeid Prawns in the Gulf of Carpentaria. *Aust. J. Mar. Freshwater Res.,* **32**, 245-251(1981).

139. Aguilera, J. A. *et al.* Determination of carbon content in steel using laser-induced breakdown spectroscopy. *Applied Spectroscopy* **46**, 1382-1387(1992).

140. Poncelow, J. *et al.* Ultrasonic determination of carbon content in uranium metal. *AIP Conf. Proc.* 1430, 1359-1365(2012).

141. MatWed. Material Property Data. Available on http://www.matweb.com/.

142. Davies, Z. G., Edmondson, J. L., Heinemeyer, A., Leake, J. R., & Gaston, K. J.. Mapping an urban ecosystem service: quantifying above-ground carbon storage at a city-wide scale. *Journal of Applied Ecology*, 48(5), 1125-1134(2011).

143. McPherson, E. G., & Simpson, J. R. *Carbon Dioxide Reduction Through Urban Forestry*. Gen. Tech. Rep. PSW-171, USDA For. Serv., Pacific Southwest Research Station, Albany, CA (1999).

144. Pataki, D. E., *et al*. Urban ecosystems and the North American carbon cycle. *Global Change Biology*, **12**, 2092-2102(2006).

145. Nowak, D. J. Atmospheric carbon-reduction by urban trees. *Journal of Environmental Management* **37**, 207–217(1993).

146. Nowak, D.J. Atmospheric carbon dioxide reduction by Chicago's urban forest. In: McPherson, E.G., Nowak, D.J., Rowntree, R.A. (Eds.), *Chicago's Urban Forest Ecosystem: Results of the Chicago Urban Forest Climate Project*. USDA Forest Service General Technical Report NE-186, Radnor, PA, pp. 83–94(1994).

147. Yang, J., McBride, J., Zhou, J., & Sun, Z. The urban forest in Beijing and its role in air pollution reduction. *Urban Forestry & Urban Greening*, **3**(2), 65-78(2005).

148. European Environment Agency (EEA). Ensuring quality of life in Europe's cities and towns. EEA Report No 5/2009. (2009)

149. Marlene Laros & Associates. The identification and prioritisation of a biodiversity network for the city of Cape Town. GISCOE (Pty) Ltd (2007).

150. Bohn, H.L. Soil absorption of air pollutants. *Journal of Environmental Quality*, **1**, 372-377(1972).

151. Pouyat, R. V., Yesilonis, I. D., & Nowak, D. J. Carbon storage by urban soils in the United States. *Journal of Environmental Quality*, **35**, 1566-1575(2006).

152. Pouyat, R.V., Groffman, P., Yesilonis, I. & Hernandez, L. Soil carbon pools and fluxes in urban ecosystems. *Environmental Pollution*, **116**, S107-S118(2002).

153. Strohbach, M. W., Arnold, E., & Haase, D. The carbon footprint of urban green space—a life cycle approach. *Landscape and Urban Planning*, **104**, 220-229(2012).

154. Singh, S., & Bakshi. B.R. Enhancing the reliability of C&N accounting in economic activities: Integration of bio-geochemical cycles with Eco-LCA. In IEEE symposium on sustainable systems and technology, IEEE-ISSST, 16–19 May, Washington, DC (2010).

155. Singh S, & Bakshi B R. Accounting for Emissions and Sinks from the Biogeochemical Cycle of Carbon in the US Economic Input-Output Model. *Journal of Industrial Ecology* **18**(6), 818–828(2014).

156. Chester M, Pincetl S & Allenby B. Avoiding unintended tradeoffs by integrating life-cycle impact assessment with urban metabolism *Curr. Opin. Environ. Sustain*. **4**, 451–457 (2012).

157. Swiss Centre for Life Cycle Inventories. Ecoinvent Version 2.01 (Switzerland: Swiss Centre for Life Cycle Inventories) (2007).

158. Leontief, W. Input-output economics. 2nd ed. Oxford, UK: Oxford University Press (1986).

159. Minx J C, Wiedmann T, Wood R, et al. Input–output analysis and carbon footprinting: an overview of applications. *Economic Systems Research*, 21(3): 187-216 (2009).

160. Miller, R.E., Blair, P.D. Input-output analysis: foundations and extensions. Cambridge

University Press, 2009, pp 348.

161. Goicoechea, A. and D.R. Hansen. An input–output model with stochastic parameters for economic analysis. *AIIE Transactions*, 10, 291–295 (1978).

162. Lenzen, M. Errors in conventional and input–output-based life-cycle inventories. *Journal of Industrial Ecology*, 4, 127–148 (2001).

163. Lenzen M, Wood R, Wiedmann T. Uncertainty analysis for multi-region input–output models– a case study of the UK's carbon footprint. *Economic Systems Research*, 22(1): 43-63 (2010).
